# FOXD1-dependent MICU1 expression regulates mitochondrial activity and cell differentiation

**DOI:** 10.1038/s41467-018-05856-4

**Published:** 2018-08-29

**Authors:** Santhanam Shanmughapriya, Dhanendra Tomar, Zhiwei Dong, Katherine J. Slovik, Neeharika Nemani, Kalimuthusamy Natarajaseenivasan, Edmund Carvalho, Christy Lu, Kaitlyn Corrigan, Venkata Naga Srikanth Garikipati, Jessica Ibetti, Sudarsan Rajan, Carlos Barrero, Kurt Chuprun, Raj Kishore, Salim Merali, Ying Tian, Wenli Yang, Muniswamy Madesh

**Affiliations:** 1 Department of Medical Genetics and Molecular Biochemistry, Lewis Katz School of Medicine at Temple University, Philadelphia, PA 19140 USA; 2 Center for Translational Medicine, Lewis Katz School of Medicine at Temple University, Philadelphia, PA 19140 USA; 3 Institute for Regenerative Medicine, University of Pennsylvania, Philadelphia, PA 19104 USA; 4 Department of Pharmaceutical Sciences, Temple University, Philadelphia, PA 19140 USA; 5 Department of Pharmacology, Lewis Katz School of Medicine at Temple University, Philadelphia, PA 19140 USA; 6 Department of Medicine and Nephrology, Center for Precision Medicine, University of Texas Health San Antonio, San Antonio, TX 78229 USA

## Abstract

Although many factors contribute to cellular differentiation, the role of mitochondria Ca^2+^ dynamics during development remains unexplored. Because mammalian embryonic epiblasts reside in a hypoxic environment, we intended to understand whether _m_Ca^2+^ and its transport machineries are regulated during hypoxia. Tissues from multiple organs of developing mouse embryo evidenced a suppression of MICU1 expression with nominal changes on other MCU complex components. As surrogate models, we here utilized human embryonic stem cells (hESCs)/induced pluripotent stem cells (hiPSCs) and primary neonatal myocytes to delineate the mechanisms that control _m_Ca^2+^ and bioenergetics during development. Analysis of MICU1 expression in hESCs/hiPSCs showed low abundance of MICU1 due to its direct repression by Foxd1. Experimentally, restoration of MICU1 established the periodic _c_Ca^2+^ oscillations and promoted cellular differentiation and maturation. These findings establish a role of _m_Ca^2+^ dynamics in regulation of cellular differentiation and reveal a molecular mechanism underlying this contribution through differential regulation of MICU1.

## Abstract (web summary)

Genetic ablation of Mitochondrial Ca2+ uptake protein 1 (MICU1) in mouse induces higher rates of perinatal lethality. Here the authors show that MICU1 expression is regulated by hypoxia in a FOXD1-dependent manner, establishing a cyclic switch between glycolytic and oxidative metabolism during development.

## Introduction

The molecular mechanisms and consequences of cellular hypoxia sensing factors are especially well characterized in many organisms during development and normal physiology. The initiation of these processes is rapid and involve transcriptional and posttranslational mechanisms. In higher order eukaryotes, survival is solely dependent upon the consumption of O_2_ as a substrate by respiratory chain complex IV, cytochrome c oxidase (COX). Upon hypoxia, the induction of COX4-2 subunit expression and a rapid degradation of COX4-1 subunit has been established in a reciprocal manner to adapt hypoxia. The HIF-1-dependent mitochondrial protease, LON degrades COX4-1 implying the well conserved O_2_-dependent homeostatic circuit at the mitochondrial level^1,2^. This phenomenon is analogues to hemoglobin F (HbF; α_2_γ_2_) vs hemoglobin A (HbA; α_2_β_2_) where HbF (~19 mmHg) has higher affinity for O_2_ over HbA (~27 mmHg) during development. Besides the well characterized prolyl hydroxylase family of enzymes and OXPHOS complex, ion channels have been proposed as physiological sensors of cellular hypoxia^3^. In the context of development, these events appear to be mediated through O_2_ and possibly requiring mitochondrial function. Because mitochondrial OXPHOS and energy transduction are mitochondrial Ca^2+^ (_m_Ca^2+^) dynamics dependent processes, relatively little is known about how the cyclic switch between glycolytic and oxidative metabolism is established during development.

A characteristic feature of the MCU channel activity is driven by two major forces like a steep mitochondrial membrane potential (Δ*Ψ*_m_) and cytosolic Ca^2+^ (_c_Ca^2+^) transients. A steep conductance of _m_Ca^2+^ rates suggests that the channel opens only if _c_Ca^2+^ attains the set-point (~2–3 μM)^4,5^. MCU functions as a pore subunit in that it can be regulated by a number of proteins, divalent cation and oxidants^4–22^. In recent years, MICU1, MICU2, and MICU3 proteins that have EF-hand Ca^2+^ binding motifs were shown to regulate the MCU activity^4,5,7,9,10,12–14,23,24^. Besides these, the MCU pore open modulator EMRE^15,25^ and an assembly factor MCUR1^21,26^ have been identified. Although recent advances have led to a better understanding of the proteins associated with MCU complex and its role in mitochondrial bioenergetics, a large and growing body of evidence attests to the complexity of regulation of MCU activity. Until now, the regulatory role of MICU1 has been reported to control MCU activity under resting state^4,5^. Our knowledge about the precise function of MICU1 is still very limited, especially regarding to mechanism of Ca^2+^ sensing and the regulation of MCU channel activity.

A loss-of-function mutation of MICU1 has been linked to human disease characterized by proximal myopathy, learning difficulties, and a progressive extrapyramidal movement disorder^27^. Recently, genetic deletion of MICU1 in murine model was reported to induce higher rates of perinatal lethality^4,28^, with embryos antepartum at expected Mendelian ratio^19^. Enigmatically, MICU1^−^^/−^ mice that did survive past 1 week show chorea-like movement. These data indicate the absence of MICU1 is particularly critical immediately after birth, suggesting MICU1 and thus _m_Ca^2+^ signaling to be critical mediators in embryonic to postnatal developmental transition when the demand for oxidative phosphorylation surges. Indeed, it is unknown whether MICU1 regulation of MCU-mediated Ca^2+^ uptake is essential for the transition from the relatively hypoxic environment of the placenta to the oxygenated environment after birth.

Here we show a differential regulation of MICU1, an effect that is caused by a suboptimal growth environment and present MICU1/MCU function analogues to STIM1/Orai-dependent SOCE phenomenon where STIM1 not only senses the ER Ca^2+^ threshold but also senses ROS and temperature^29–31^. Importantly, tissues from brain, heart, liver, and lung of developing mouse embryo (E13.5, and E16.5) evidenced a suppression of MCU gatekeeper, MICU1 expression with minimal regulation on other MCU complex components under hypoxic milieu. We utilized human induced pluripotent stem cells (hiPSCs) and neonatal myocytes to delineate the mechanisms that control _m_Ca^2+^ dynamics and mitochondrial bioenergetics during development. Analysis of MICU1 protein expression in parental fibroblast (GM942) and the corresponding re-programmed hiPSCs showed mere absence of MICU1 in hiPSCs. We also observed a differential regulation of MICU1 in NRVMs in response to hypoxia. Our data reveals that the constitutive accumulation of _m_Ca^2+^, mitochondria ROS, and global proteomic changes in hiPSCs is due to the loss of MCU gatekeeping effect. In glycolytic and hypoxic hiPSCs and neonatal myocytes, MICU1 expression is under the repression of Foxd1 transcription factor. Reconstitution of MICU1 either by Foxd1 knock down or ectopic expression restores the periodic cytosolic Ca^2+^ transients that is a prerequisite for cellular differentiation. These findings reveal a distinct molecular mechanism underlying the contribution of mitochondrial function in low oxygen tension and the functional role for MICU1 in possibly sensing hypoxia.

## Results

### MICU1 expression is differentially regulated by hypoxia

**Fig. 1:**
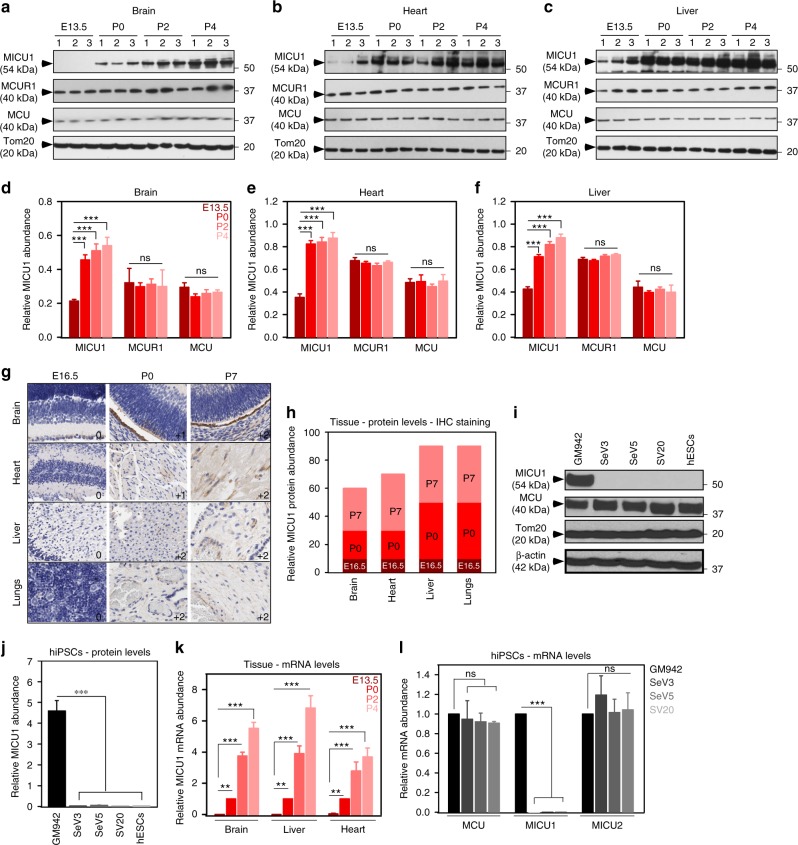
MICU1 is differentially expressed during embryonic development. **a**–**c** Representative western blot for lysates from brain (**a**), heart (**b**), and liver (**c**) harvested from embryo (E13.5), and neonates (P0, P2, and P4). The western blots were probed with antibody specific for MCU, MICU1, MCUR1, and Tom20. **d**–**f** Quantification of relative MCU, MICU1, and MCUR1 protein abundance from **a**–**c**. **g** Representative zoomed microscopic images of the tissue microarray probed with MICU1 antibody. **h** Quantification of MICU1 expression levels represented as percent MICU1 expression as a function of tissue staining. **i** Representative western blot for lysates from control fibroblasts (GM942), hiPSCs (SeV3, SeV5, and SV20), and hESCs probed with antibody specific for MCU, MICU1, Tom20, and β-actin. **j** Quantification of relative MICU1 protein abundance from **i**. **k** Quantification of MICU1 mRNA levels in different tissues (brain, liver, and heart) harvested from embryo and neonates (E13.5, P0, P2, and P4). **l** Quantification of MICU1 mRNA levels in two independent clonal hiPSCs lines (SeV3 and SV20) and one clonal line of SeV3 (SeV5). Data represents Mean ± SEM; **P < 0.01; ***P < 0.001; *n* = 3–5 (one-way ANOVA)

Having demonstrated the importance of MICU1 in human and mouse physiology^4,28^, we investigated the role of MICU1 in development. We assessed the expression profile of MCU complex components in multiple metabolically active organs including brain, heart, lungs, and liver from different mouse developmental stages (E13.5, E16.5, P0, P2, P4, and P7). The western blot analysis of the fetal and postnatal tissues with antibodies specific for MCU, MICU1, and MCUR1 showed a differential expression of MICU1 with no observable changes in other MCU complex components (Fig. 1a, f). Similarly, the immunohistochemical analysis of multiple tissues showed differential expression of MICU1 (Fig. 1g, h). As depicted P0 and P7 neonate tissues showed higher expression of MICU1 while E16.5 embryos showed lower levels of MICU1 expression (Fig. 1g, h). Because we observed a differential expression of MICU1 in developmental stages, we hypothesize the increased trans-placental oxygen levels and thus modulation of tissue oxygen content to modulate MICU1 expression. As a surrogate model to test whether MICU1 is regulated by the hypoxic microenvironment, we subjected the freshly isolated neonatal rat ventricular myocytes (NRVMs) to normoxia at various time points (48–168 h) or hypoxia for 24 h followed by reoxygenation (72 and 96 h). Western blot analysis revealed a progressive increase of MICU1 protein expression under normoxic culture condition (Supplementary Fig.1a and 1b). Remarkably, the repression of MICU1 expression was observed under hypoxia and re-oxygenation restores the MICU1 protein abundance (Supplementary Fig.1a and 1b).

Because stem and adult cells reside in their specific niches or anatomic locations that modulate cellular differentiation^32–36^, we next asked whether a differential expression of MICU1 was observed in human embryonic stem cells (hESCs). Subsequently, hiPSCs have replaced the well-known hESCs as they are generated in an individual-matched manner. To assess the expression of MICU1 in hiPSCs, two independent clonal hiPSC lines (SeV3 and SeV5) derived from the same skin fibroblasts (GM942), one hiPSC line: SV20^37^ generated from blood and the hESC line H9 were used (Supplementary Fig.1c-1e). Western blot analysis indicated high levels of MICU1 protein expression in control fibroblasts (GM942) while hiPSCs and hESCs showed low abundance of MICU1 protein (Fig. 1i, j, Supplementary Fig. 1f and 1g). The decrease in MICU1 protein expression was consistent with decreased mRNA abundance in the embryonic tissues (Fig. 1k) and hiPSCs (Fig. 1l).

### Foxd1 mediated transcriptional regulation of MICU1

**Fig. 2:**
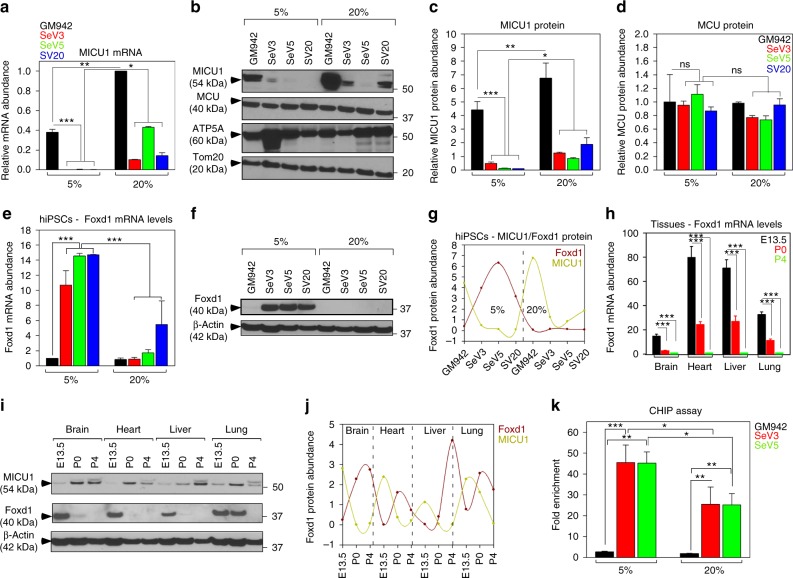
MICU1 is transcriptionally regulated by Foxd1 under hypoxia. **a** Quantification of MICU1 mRNA abundance in hiPSCs exposed to normoxia (20% O_2_) and hypoxia (5% O_2_). **b** Representative western blot for lysates from control fibroblasts and hiPSCs grown under hypoxic/normoxic conditions and probed with antibody specific for MICU1, MCU, ATP5A, and Tom20. **c**, **d** Quantification of relative MICU1 (**c**) and MCU (**d**) protein abundance in hiPSCs grown under hypoxic/normoxic conditions. **e** Quantification of Foxd1 mRNA abundance in hiPSCs under normoxia/hypoxia. **f** Representative western blot for lysates from control fibroblasts and hiPSCs grown under normoxic/ hypoxic conditions and probed with antibody specific for Foxd1 and β-actin. **g** Quantification of relative protein abundance of Foxd1 and MICU1 quantified from **f** and **b**. **h** Quantification of Foxd1 mRNA abundance in various tissues (brain, heart, liver, and lung) harvested from different stages of embryonic development. **i** Representative western blot for lysates from brain, heart, liver, and lung harvested from embryos/neonates and probed with antibodies specific for MICU1, Foxd1, and β-actin. **j** Quantification of relative protein abundance of Foxd1 and MICU1 quantified from **i**. **k** ChIP-assay was performed in hiPSCs exposed to normoxia and hypoxia. Antibody specific for Foxd1 was used to immunoprecipitate the chromatin and the fold enrichment of *micu1* promoter relative to the matched input control was quantified by qRT-PCR. Bar represents Mean ± SEM, **P < 0.01, *P < 0.05, ***P < 0.001; *n* = 3–5 (One-Way ANOVA)

Next, we asked whether differential regulation of MICU1 expression is transcriptionally controlled under hypoxic micro-environment. hiPSCs or NRVMs were subjected to both hypoxia (5% O_2_) or normoxia (20% O_2_) and MICU1 expression was evaluated. As expected MICU1 mRNA and protein abundance were lower under hypoxic culture condition in hiPSCs (Fig. 2a–c, Supplementary Fig. 1h). Strikingly, MICU1 mRNA levels by qPCR analysis show an increased expression of MICU1 transcript in hiPSC clonal lines when cultured at normoxic condition (Fig. 2a). In line with increased MICU1 transcript, hiPSCs exhibited elevated MICU1 protein levels under normoxia with no observable changes in MCU protein (Fig. 2b, d, Supplementary Fig. 1h). Similar to hiPSCs, MICU1 mRNA abundance was observed in NRVMs exposed to normoxia (Supplementary Fig. 1i). Analysis of the promoter sequence of 411 genes upregulated in reprogrammed iPSCs revealed two transcription factors Foxd1 and Foxo1 to be overrepresented^38^. Though Foxo1 is a well-known regulator of longevity, stress response^39,40^, and essential for maintaining pluripotency of ESCs^41^, Foxd1 has been shown to function in the development of kidney^42–44^. In line with these findings, we investigated the expression levels of Foxd1 in control fibroblasts and hiPSCs. hiPSCs have suggestively increased levels of Foxd1 that was reciprocal to the MICU1 expression (Supplementary Fig. 1j and 1k). Because we observed MICU1 to be transcriptionally regulated under normoxic and hypoxic conditions (Fig. 2a, c), we next investigated whether Foxd1 is the major transcriptional regulator for MICU1 under conditions of hypoxia. Foxd1 mRNA abundance was determined in hiPSCs under conditions of normoxia/hypoxia. Foxd1 transcript levels reflected the changes in Foxd1 protein abundance (Fig. 2e, g, Supplementary Fig. 1l). Of interest, hiPSCs maintained at 20% oxygen showed striking decrease in Foxd1 mRNA (Fig. 2e) and protein levels (Fig. 2f, g, Supplementary Fig. 1l). In addition, we observed a reciprocal relationship between the Foxd1 /MICU1 mRNA (Fig. 2h) and protein levels (Fig. 2i, j, Supplementary Fig. 1m) in embryonic/neonatal tissues that were correlated with hypoxia and normoxia conditions. Taken together, these results indicate Foxd1 to transcriptionally repress MICU1 expression under condition of hypoxia.

Next, we asked whether *micu1* promoter have a binding site for Foxd1. Bioinformatic analysis of *micu1* promoter sequence predicted a conserved putative binding site for Foxd1 at −302–297 base pairs (bp) (TCAACA). To investigate the functional interactions between the *micu1* promoter and Foxd1 during hypoxia, we performed a ChIP assay in hiPSCs under conditions of hypoxia/normoxia. Nuclear extracts from hiPSC clones maintained in hypoxic conditions showed increased Foxd1 binding to the *micu1* promoter (Fig. 2k), which parallels decreased MICU1 expression (Fig. 2a, b). Additionally, to ensure the consistency of increased Foxd1 binding to *micu1* promoter, we performed ChiP assay in human pulmonary microvascular endothelial cells (HPMVECs) under conditions of hypoxia/normoxia. Similar to hiPSCs, HPMVECs exposed to hypoxia display increased binding of Foxd1 to the *micu1* promoter and reoxygenation restored Foxd1 binding (Supplementary Fig. 1n). After confirming the ability to replicate the phenomenon of Foxd1 binding to *micu1* promoter in different cell types, we generated luciferase reporter constructs of *micu1* promoter that harbors the Foxd1 binding site (Supplementary Fig. 1o). The luciferase constructs were expressed in HPMVECs and 48 h post-transfection, luciferase activity was measured after exposure of HPMVECs to hypoxia and reoxygenation. HPMVECs expressing *micu1* promoter construct (-302-297 bp) exhibited differential expression of luciferase in response to hypoxia and reoxygenation (Supplementary Fig. 1p). Conversely, in cells expressing *micu1*^Δ-302–297^ the luciferase expression was not regulated by hypoxia, indicating Foxd1- mediated transcriptional repression of MICU1 during hypoxia. Further, to systematically show Foxd1 to be the transcriptional repressor for MICU1, we adopted RNAi based knockdown of Foxd1 in hiPSCs clonal lines. In agreement with the promoter and ChIP analysis, hiPSCs transfected with Foxd1 siRNA restored MICU1 expression levels, validating Foxd1-mediated transcriptional repression of MICU1 expression (Supplementary Fig. 1q and 1r).

### Loss of MICU1 regulates _m_Ca^2+^ uptake and bioenergetics

**Fig. 3:**
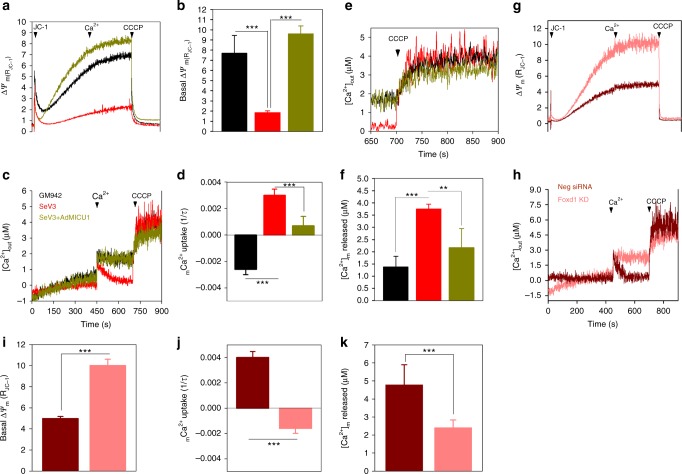
Loss of MICU1 in hiPSCs augment MCU-mediated Ca^2+^ uptake and overloads matrix Ca^2+^. An equal number of fibroblasts (GM942), hiPSCs (SeV3), and SeV3 expressing MICU1cells (6 × 10^6^) were permeabilized with digitonin (40 μM), loaded with JC-1 (800 nM) and Fura-FF (1 μM) and changes in Δ*Ψ*_m_ and _m_Ca^2+^ uptake was measured using a multi-wavelength excitation dual-wavelength emission spectrofluorimeter. **a** Mean traces of Δ*Ψ*_m_ in fibroblasts and hiPSCs. **b** Quantification of basal Δ*Ψ*_m_ before addition of an extramitochondrial Ca^2+^ bolus. **c** Mean traces of [Ca^2+^]_out_ measured in permeabilized fibroblasts and hiPSCs. **d** Quantification of the rate of _m_Ca^2+^ uptake as a function of decrease in bath Ca^2+^ fluorescence after an extramitochondrial Ca^2+^ pulse (1 μM). **e** Mean traces of [Ca^2+^]_out_ after addition of CCCP. **f** Quantification of total _m_Ca^2+^ released after the addition of CCCP as a function of increase in bath Ca^2+^ fluorescence. Foxd1 knock down restores MICU1 gate keeping effect. **g**–**k** SeV3 cells were transiently transfected with control and Foxd1 siRNA and 48 h post transfection, Δ*Ψ*_m_ and _m_Ca^2+^ uptake was measured simultaneously in permeabilized cells. **g** Mean traces of Δ*Ψ*_m_ in control (Neg siRNA) and Foxd1 KD hiPSCs. **h** Mean traces of [Ca^2+^]_out_ measured in permeabilized control (Neg siRNA) and Foxd1 KD hiPSCs. **i** Quantification of basal Δ*Ψ*_m_ before addition of an extramitochondrial Ca^2+^ bolus. **j** Quantification of the rate of _m_Ca^2+^ uptake as a function of decrease in bath Ca^2+^ fluorescence after an extramitochondrial Ca^2+^ pulse (1 μM). **k** Quantification of total _m_Ca^2+^ released after the addition of CCCP. Data represents Mean ± SEM; **P < 0.01, ***P < 0.001; *n* = 4–6 (One-way ANOVA)

Because, MICU1 is the gate keeper for MCU that determines the set-point for MCU-mediated _m_Ca^2+^ uptake, and prevent _m_Ca^2+^ overload, we next asked whether the repression of MICU1 in hiPSCs result in constitutive MCU-mediated _m_Ca^2+^ accumulation even at [Ca^2+^]_i_ below the threshold (>3 μM)^5^. We performed simultaneous measurement of _m_Ca^2+^ uptake and mitochondrial membrane potential (Δ*Ψ*_m_) in permeabilized fibroblast and hiPSC clone, SeV3. Digitonin permeabilized cells were bathed in intracellular-like media (ICM) containing thapsigargin (Tg) to prevent Ca^2+^ uptake through SERCA and loaded with FuraFF (bath Ca^2+^ indicator) and JC-1 (Δ*Ψ*_m_ indicator) (Fig. 3a, c). A single bolus of extramitochondrial Ca^2+^ pulse (1 μM) within the functional inhibitory range of MICU1, was added and the rate of _m_Ca^2+^ uptake was measured as a function of decrease in bath Ca^2+^ fluorescence. SeV3 was able to rapidly clear 1 μM Ca^2+^ pulse indicating loss of MICU1 inhibition on MCU activity. Conversely, fibroblasts did not clear the 1 μM Ca^2+^ and evidenced intact MICU1 function (Fig. 3c, d). An uncoupler CCCP was added at 750 s to depolarize the mitochondrial membrane and release accumulated matrix Ca^2+^. Consistent with the absence of MICU1 in SeV3, we observed constitutive activation of MCU and increased _m_Ca^2+^accumulation (Fig. 3e, f). Next, we asked whether excessive accumulation of _m_Ca^2+^ have an impact on Δ*Ψ*_m_. We observed increased accumulation of _m_Ca^2+^ to result in decreased Δ*Ψ*_m_ further substantiating MCU is relieved from MICU1 gatekeeping effect (Fig. 3a, b; Supplementary Fig. 2a and 2b). Next, we restored MICU1 expression in hiPSCs either by ectopic expression of MICU1 using adenovirus or by knocking down Foxd1. We measured _m_Ca^2+^ uptake and Δ*Ψ*_m_ in MICU1 restored SeV3 (Fig. 3). Either ectopic expression of MICU1 (Fig. 3a, f) or silencing Foxd1 (Supplementary Fig. 1q and 1r; Fig. 3g, k) re-established the gatekeeping effect on MCU-mediated Ca^2+^ uptake and maintained Δ*Ψ*_m_. Next, we asked whether constitutive activation of MCU and excessive accumulation of _m_Ca^2+^ results in increased mROS generation. In line with constitutive MCU activation, hiPSCs showed elevated mROS compared to control fibroblasts (Supplementary Fig. 2c and 2d).

Because we were able to mimic the regulation of MICU1 by hypoxia in NRVMs (Supplementary Fig. 1a and 1b), we then performed the simultaneous measurement of _m_Ca^2+^ uptake and Δ*Ψ*_m_ in permeabilized NRVMs exposed to hypoxia and reoxygenation (Supplementary Fig. 2e-2j). Consistent with the rapid clearance of 1 μM Ca^2+^ bolus by hiPSCs, NRVMs exposed to hypoxia also show a constitutive activation of MCU with a functional phenotype similar to loss of MICU1^5^. Matrix Ca^2+^ is known to activate the dehydrogenases and increase the mitochondrial oxygen consumption rate (OCR). On the other hand, excessive accumulation of matrix Ca^2+^ results in mitochondrial permeability transition pore opening and bioenergetics crisis. Next, we assessed the cellular bioenergetics by exposing NRVMs sequentially to the inhibitors of the complex activity (Supplementary Fig. 2k). Consistent with _m_Ca^2+^ overload, basal, and maximal OCR were significantly reduced in NRVMs exposed to hypoxia (Supplementary Fig. 2l-2n). Additionally, NRVMs exposed to hypoxia show increased proton leak (Supplementary Fig. 2n), further validating uncoupled Ca^2+^ activated OXPHOS during hypoxia.

### Reconstitution of MICU1 establish _i_Ca^2+^ transients in hiPSCs

**Fig. 4:**
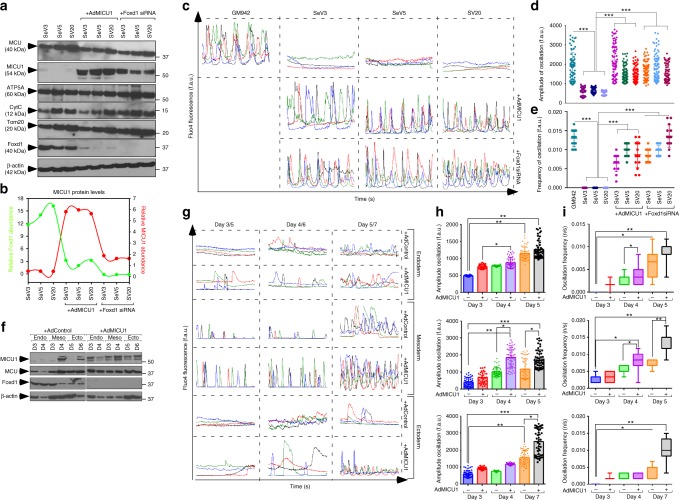
Reconstitution of MICU1 in hiPSCs establishes _i_Ca^2+^ transients and modulates lineage-specific cell differentiation. **a** Representative western blot for lysates from hiPSCs with and without MICU1 expression. The western blots were probed with antibody specific for MICU1, MCU, ATP5A, Cytochrome C, Foxd1, Tom20, and β-actin. **b** Quantification of relative protein abundance of Foxd1 and MICU1. **c** Traces of spontaneous cytosolic Ca^2+^ oscillations in control fibroblasts and hiPSCs with and without MICU1 expression. Intact cells were loaded with fluo4 and spontaneous Ca^2+^ oscillations were observed at 488 nm. **d** Quantification of peak fluo-4 fluorescence. **e** Quantification of the frequency of _c_Ca^2+^oscillations. **f** Representative western blot for lysates from hiPSCs induced for lineage specific differentiation for the specified days with and without MICU1 expression. The western blots were probed with antibody specific for MICU1, MCU, Foxd1, and β-actin. **g** Traces of spontaneous _c_Ca^2+^ oscillations in hiPSCs upon induction of lineage specific differentiation with and without MICU1 expression. Ca^2+^ transients were measured at different days of differentiation: Day 3, 4, and 5 for endoderm and mesoderm; Day 5, 6, and 7 for ectoderm. **h** Quantification of peak fluo-4 fluorescence. **i** Quantification of the frequency of _c_Ca^2+^oscillations. Data indicate quantified peak fluorescence/frequency of oscillation. Mean ± SEM is represented in the figures; **P < 0.01, *P < 0.05, ***P < 0.001; *n* = 3–4 (One-Way Anova) individual experiments. Dots represents mean data from group of single cells

Intracellular Ca^2+^ (_i_Ca^2+^) transients are maintained by the simultaneous interplay of counteracting processes, the on and off mechanisms^45,46^. _i_Ca^2+^ oscillations play a pivotal role in intracellular signaling and is also a control oscillatory regime for many cellular processes. Recent studies have shown hiPSCs to have functional IP_3_-regulated intracellular Ca^2+^ stores and store-operated Ca^2+^ entry (SoCE) for refiling of their stores. They are sensitive to stimulation with ATP, histamine, and platelet-derived growth factor. It has long been studied and accepted that a fine regulation of _i_Ca^2+^ transients are maintained by mitochondria, that has a unique ability to decode and transduce Ca^2+^ signals into an energy output (ATP). Since we observed low abundance of MICU1 in hiPSCs (Fig. 1i, 4a, b, Supplementary Fig. 3a), we next asked whether the loss of MICU1 impairs _m_Ca^2+^ buffering and thus alters the _i_Ca^2+^ oscillatory phenotype. Both control fibroblasts, hiPSCs (SeV3, SeV5, and SV20), and hESCs were loaded with Fluo-4 and spontaneous _i_Ca^2+^ oscillations were measured. Fibroblasts showed an oscillatory phenotype, while hiPSCs and hESCs exhibited no _i_Ca^2+^ oscillations implicating perturbed _i_Ca^2+^ buffering possibly by mitochondria (Fig. 4c, e and Supplementary Fig. 3b and 3c). To substantiate the loss of MICU1 as a cause for perturbed _i_Ca^2+^ transients, we reconstituted MICU1 either by ectopic expression or by knocking down Foxd1 (Fig. 4a, b, Supplementary Fig. 3a) in hiPSCs. The reconstitution of MICU1 re-established the _i_Ca^2+^ oscillatory phenotype corroborating MICU1’s function in maintaining cellular Ca^2+^ transients (Fig. 4c, e and Supplementary Fig. 3b and 3c).

### _i_Ca^2+^ transients modulate cell differentiation

The link between the spatiotemporal pattern of _i_Ca^2+^ transients and expression of developmental genes is a key phenomenon, and that the frequency as well as the amplitude of Ca^2+^ transients is important for the regulation of gene expression^47^. The features of Ca^2+^-dependent expression are not only restricted to cells but also crucial in embryo development. During *Xenopus* embryonic development, the expression of early neural genes ZiC3 and geminin were downregulated by Ca^2+^ transients in a spatiotemporal pattern which was confirmed by the blockade of l-type Ca^2+^ channels^48,49^. Given the restoration of the Ca^2+^ oscillatory phenotype upon MICU1 expression in hiPSCs, we asked whether reconstitution of MICU1 is accompanied by a switch in hiPSCs to a more differentiated phenotype. To this end, we mapped the proteomes of fibroblasts (GM942), and hiPSCs with and without MICU1 expression (SeV3, and SeV3 + AdMICU1) using label-free proteomics (GeLC-MS/MS). 6220 proteins were identified when analyzing the fold-change expression ratios comparing fibroblasts and hiPSCs. Of these, 662 proteins were expressed exclusively in fibroblasts and control hiPSCs, 985 in fibroblasts and hiPSCs expressing MICU1, and 1202 proteins in common (Supplementary Fig. 3d). This data infers the differential expression of a relatively small number of proteins in cells that express MICU1. Gene Ontology (GO) enrichment analysis revealed that cell cycle and related processes including DNA replication, nucleotide biosynthesis, cell proliferation, organ development, and function genes were overrepresented in MICU1 reconstituted hiPSCs (Supplementary Fig. 3e). Also, the mapping of fibroblasts and hiPSCs revealed enrichment of developmental markers (embryonic, tissue, and cardiovascular) by MICU1 expression (Supplementary Fig. 3f and 3g). Indeed, protein-protein interaction network analysis highlighted major Ca^2+^-cellular signaling pathways including the MAPK and NF-κB signaling proteins being expressed at elevated levels in hiPSCs expressing MICU1 (Supplementary Fig. 3h). Additionally, the epithelial to mesenchymal transition (EMT) pathway proteins, N-Cadherin, Vimentin, MMPs, nuclear β-catenin, and fibronectin were enriched in hiPSCs expressing MICU1 (Supplementary Fig. 3i and 3j). Of the transcriptional factors involved in EMT, ZEB was highly elevated in hiPSCs expressing MICU1. Because, hiPSCs share similarity to ESCs and undergo same trend of EMT during differentiation, we asked whether the ectopic expression of MICU1 change the pluripotent nature of hiPSCs. Surprisingly, MICU1 expression did not alter the mRNA levels of OCT4, and NANOG (Supplementary Fig. 3k) when cells were cultured under pluripotency-maintaining conditions. EMT transcript Zeb2 was indicated to modulate cell-fate decision during the transformation of ESCs to primary germ layer differentiation^50,51^. Like gastrulation where most of the cells undergo EMT, we expect ectopic expression of MICU1 in hiPSCs to result in a hybrid EMT that enable cells to identify an external signal and acquire maximum cellular transition. It has been known that a fully transformed mesenchymal fate to be associated with mesoderm development, but neural crest delamination in the ectoderm or partial endoderm formation are also consequences of EMT^52,53^.

Having observed the pluripotency markers to remain unaltered in hiPSCs expressing MICU1 and EMT pathway proteins to be over-represented, we asked whether ectopic expression of MICU1 regulate iPSC-derived early lineage specification. SV20 hiPSCs cultured in pluripotency-maintaining media were infected with AdMICU1. 48 h post-infection, cells were replated and induced for early lineage commitment by separately applying cytokines that specify each of the three germ layers: endoderm, mesoderm, and ectoderm. While the ectopic expression of MICU1 (Fig. 4f, Supplementary Fig. 4a) significantly upregulated genes of the endoderm lineage (Supplementary Fig. 4b), but had little or no effect on mesoderm or ectoderm genes (Supplementary Fig. 4c and 4d).

We next asked whether MICU1 expression is restored in hiPSCs as they differentiate into different cell lineages. The differentiation of cells was accompanied by the up-regulation of MICU1 protein (Fig. 4f, Supplementary Fig. 4a) and restored the _i_Ca^2+^ transients which is a prerequisite for cell differentiation. Spontaneous Ca^2+^ oscillation was observed in hiPSCs upon induction of lineage specific differentiation, with observable stimulation in endodermal and mesodermal lineages (Fig. 4g, i). Next, we asked whether ectopic expression of MICU1 cause a leftward shift (early) in the induction of Ca^2+^ oscillation and could explain the early lineage selection. The ectopic expression of MICU1 resulted in a significant difference in the amplitude of Ca^2+^ oscillation in mesoderm and endoderm, while the ectodermal Ca^2+^ oscillation remain unaffected (Fig. 4g, i).

### Expression of MICU1 promotes cardiomyocyte maturation

**Fig. 5:**
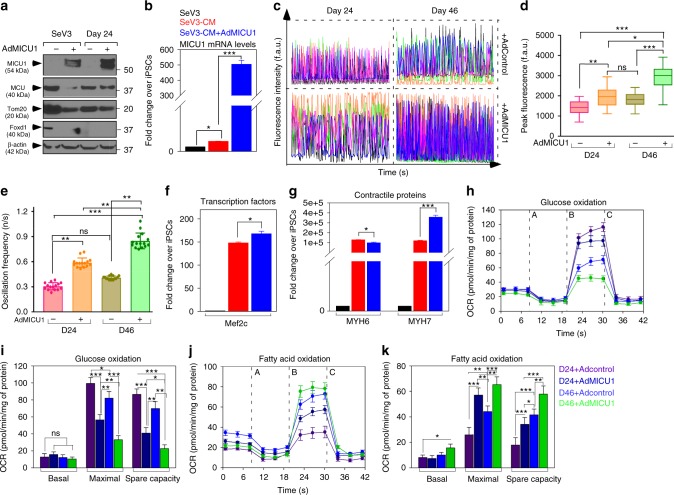
MICU1 expression promotes iPSC-Cardiomyocyte maturation. **a** Representative western blot for lysates from SeV3 and SeV3-derived cardiomyocytes (SeV3-CMs) with and without MICU1 expression. The western blots were probed with antibody specific for MICU1, MCU, Foxd1, Tom20, and β-actin. **b** Quantification of the MICU1 mRNA abundance in SeV3 and day 24 SeV3-CMs. **c** Traces of spontaneous cytosolic Ca^2+^ oscillations in SeV3-CMs with and without MICU1 expression at day 24 and day 46 post-differentiation. **d** Quantification of peak fluo-4 fluorescence. **e** Quantification of the frequency of _c_Ca^2+^oscillations. Data indicate Mean ± SEM; **P < 0.001, ***P < 0.001; *n* = 3–4 independent experiments. Dots represents data from group of single cells. **f** Quantification of mRNA abundance of the early cardiac transcription factor Mef2c in SeV3-CMs with or without MICU1 expression at day 24 of differentiation. **g** Quantification of mRNA abundance of the cardiac contractility proteins, MYH6, and MYH7 in SeV3-CMs with or without MICU1 expression at day 24 of differentiation. Data indicate Mean ± SEM; *P < 0.05, ***P < 0.001; *n* = 3–4. **h**, **j** Measurement of oxygen consumption rate (OCR) in SeV3-CMs with or without MICU1 expression using glucose (glucose oxidation) (**h**) or carnitine (fatty acid oxidation) (**j**) as substrate (glucose oxidation). After basal OCR measurement, oligomycin (A), FCCP (B), and rotenone + Antimycin A (C) were added as indicated. **h**, **j** Representative traces of OCR in SeV3-CMs. **i**, **k** Quantification of basal and maximal OCR, and spare capacity in SeV3-CMs. Data indicate Mean ± SEM; *P < 0.05, **P < 0.01, ***P < 0.0001; *n* = 3

EMT has been identified as one of the first steps of cardiac differentiation^52–54^ with Ca^2+^ as an essential signal integrator for differentiation of ESCs to functional cardiomyocytes. Because, we observed EMT pathway proteins to be over-represented during ectopic expression of MICU1, to further evaluate the role of MICU1 in cardiomyocyte differentiation and maturation, we generated hiPSCs-derived cardiomyocytes (hiPSC-CMs), using established protocol which results in immature myocytes with mixed subtypes^55,56^ and ectopically expressed MICU1 in these cells for 5 days. Not surprisingly, hiPSCs-CMs had low levels of MICU1 (Fig. 5a, b, Supplementary Fig. 5a) which correlated with the MICU1 levels of rat neonatal myocytes (Supplementary Fig. 5b-5d). In effect the differentiation-day 24 hiPSCs-CMs exhibited immature spontaneous _c_Ca^2+^ transients (Fig. 5c, e) resembling neonatal (P0) cardiomyocytes (Supplementary Fig. 5f and 5g). Whereas the recorded transients were consistent in amplitude and frequency in the hiPSCs-CMs expressing MICU1 (Fig. 5c-e). Analysis of spontaneous _i_Ca^2+^ transients at day 46 show no observable changes between day 24 and 46 in the frequency and the amplitude. Conversely, ectopic expression of MICU1 show an increase in the frequency and amplitude of _i_Ca^2+^ transients mimicking NRVMs isolated from neonates (Fig. 5c-e and Supplementary Fig. 5f and 5g). Surprisingly, quantitative qPCR analysis showed a more differentiated phenotype in MICU1 expressing d24 hiPSC-CMs, where expression of genes encoding cardiac transcription factor, Mef2c (Fig. 5f) and cardiac structural and contractile proteins: MYH6 and MYH7 (Fig. 5g) were significantly regulated when compared to hiPSCs-CMs. This cardiac transcript profile of the hiPSCs-CMs expressing MICU1 is indicative of maturing myocytes^57–59^.

Despite the very high energy demand, the adult heart has essentially no energy reserves and continuously produce ATP to meet the work load. Mitochondrial oxidative phosphorylation provides over 90% of the energy demand with fatty acid being the major source of oxidative metabolism. In contrast to the mature cardiomyocyte, energy metabolism during myocyte development differ quite dramatically. In the early embryonic stage, the cardiomyocyte precursor seems to be dependent on glycolysis as a source of energy. However successful transition of the ESCs to cardiomyocytes requires a switch from glycolytic metabolism to mitochondrial oxidative phosphorylation. Maintaining high glycolytic rates facilitate the proliferative state of the developing cardiomyocyte, whereas increase in mitochondrial oxidative capacity marks a more terminally differentiated myocyte. To determine if modulation of MICU1 levels play a role in metabolic phenotype, OCR was measured in hiPSCs-CMs with or without ectopic expression of MICU1 using glucose (Fig. 5h) or carnitine (Fig. 5j) as substrate. The ectopic expression of MICU1 markedly increased the contribution of fatty acid oxidation to overall cardiac energetics of hiPSCs-CMs (Fig. 5j, k) approaching levels observed in myocytes isolated from P5 (Supplementary Fig. 5h-5l). The increase in fatty acid oxidation was paralleled by the decrease in glucose oxidation (Fig. 5h, i). Our data provide an experimental confirmation that mitochondrial calcium signaling plays an important role in cardiac development and maturation.

## Discussion

Since mitochondria establish a huge thermodynamic force, the ions and metabolites that are fluxed into mitochondria are used for metabolic cascades. In parallel, over 90% of the cellular O_2_ is consumed for ATP generation. Having established _m_Ca^2+^ dynamics, oxygen consumption and energy production are well coupled sequential cellular processes evolutionarily, there must be several check points that could be sensed and controlled during the developmental processes. In the developing heart, the cardiac energetic demand increases with increasing cardiac performance required for embryonic growth^60–62^. The morphological changes occurring between an early embryonic stage (E9.5) and late embryonic stage (E13.5) in the heart coincide with significant changes in mechanical load, contractile force generation, and blood flow. These epigenetic factors including changes in intracardiac hemodynamics and mechanical stress could impact mitochondrial maturation and myocyte differentiation. A study establishes mechanical stretch to promote ES cell differentiation into myocytes^63^. Evidently, between E8.5 and E10 the pO_2_ in the mouse heart is lower than 10 mm Hg^64^. Because the embryos at this stage have lower O_2_ levels due to lack of placental-embryonic circulation^64^, we expect absence of MICU1 in early embryonic myocytes due its transcriptional regulation. We also expect a restoration of MICU1 in myocytes as the heart establishes the placental-embryonic circulation after E10. Such a controlled regulation of MICU1 expression should influence cellular differentiation and maturation. While MICU1 reconstitution promoted myocytes maturation, we did not observe a significant effect on early lineage differentiation. One possibility is that the inductive cues that drive the initial lineage commitment are very dominant and are overriding the impact of ectopic MICU1. Further analysis of the effect of MICU1 on lineage specification will need to be carried out to address this possibility. Nonetheless, our study is the first to establish the role of mitochondrial calcium signaling during development and cellular differentiation.

Our study also have implications on maturation of hiPSC-derived cardiomyocytes and other cell types. It is well appreciated that a common limitation of using hiPSC-derived cell lineages for disease modeling is the immaturity of the cells compared to their fully developed in vivo counterparts^65,66^. Functional maturation is a difficult obstacle to overcome in vitro despite long term culture and is the subject of intense investigation. Our finding that modulation of _m_Ca^2+^ dynamics in immature cardiomyocytes promotes a more mature phenotype suggests that ectopic expression of MICU1 in newly formed iPSC-CMs may be utilized in combination with other favorable engineered microenvironments to generate more mature tissues in vitro. This approach may also potentially be applied to other metabolically active hiPSC-derived lineages such as neurons and hepatocytes.

An intriguing question that arises from our study is how embryonic myocytes compensate for the phenotypes of ablated MICU1 expression resulting in constitutive MCU activation, accumulation of matrix Ca^2+^ and elevated mROS generation. It is possible that there could be a compensatory mechanism that may result in the opening of the mitochondrial permeability transition pore (mPTP) in early embryonic heart^67^. Because MCU-mediated Ca^2+^ uptake (anterograde) and mROS generation (reterograde) are often interdependent phenomenon, we believe a complex role for both _m_Ca^2+^ and redox signaling during embryonic development. In the early embryonic stage _m_Ca^2+^-mediated mROS is essential for initial commitment of progenitor cells towards differentiated cell lineage through regulated _m_Ca^2+^ and mROS. Having described how the hypoxic signals could be decoded as ion regulatory signals in maintaining developmental plasticity, thereby facilitating adaptation to stress conditions and to allow a well-integrated tissue repair response, future studies are warranted to better understand the role of MCU-mediated Ca^2+^ signaling during development.

## Methods

### Animals and cell culture

Wild type C57BL/6 mice were maintained in our animal facility in accordance and with approval from Institutional animal care and use committee. Tissues including brain, heart, and liver were harvested from deeply anesthetized mice with a mixture of xylazine (40 mg/kg) and ketamine (80 mg/kg). The harvested tissues were perfused with PBS and stored until further use.

Ventricular cardiomyocytes from embryos (E9.5, and E13.5) or neonates (P0, P1, and P4) heart (NRVMs) were prepared as previously described^68^. Embryonic and neonatal mycoytes were cultured in Ham’s F-10 supplemented with 5% fetal bovine serum (FBS) and penicillin/streptomycin (100 U/ml) at 37 °C in a 95% air/5% CO_2_ humidified atmosphere for 4 days. Human microvascular pulmonary endothelial cells (HPMVECs) was purchased from American Type Culture Collection (Manassas, VA, USA) and cultured in DMEM-high glucose media supplemented with 20% FBS and penicillin/streptomycin (100 U/ml) at 37 °C in a 95% air/5% CO_2_ humidified atmosphere.

### hiPSCs derivation and culture of hiPSCs/hESCs

Experiments involving the use of hESCs and hiPSCs have been approved by the University of Pennsylvania Embryonic Stem Cell Research Oversight Committee. Human primary fibroblasts (GM00942) from an apparently healthy 5 year old female Caucasian donor were obtained from Coriell Institute which operates the National Institute of General Medical Sciences (NIGMS) Human Genetic Cell Repository. The NIGMS Informed Consent was obtained from patient at time of sample submission. Coriell also obtained a Certificate of Confidentiality from the National Institutes of Health to help ensure patient’s privacy. iPSC cell lines iPS-GM942-SeV3 and iPS-GM942-SeV5 were derived from GM942 fibroblasts using Sendai Viral reprogramming vectors (Cytotune^TM^, Thermofisher Scientific) according to manufacturer’s instructions. The iPSC-SV20 cell line has been described previously^37^ and has been deposited at WiCell Institute under the name PENN123i-SV20. The hESC cell line H9 was purchased from WiCell Institute. hiPSCs/hESCs were maintained on Geltrex (Thermofisher Scientific)—coated plates in either mTeSR1 (Stem Cell Technologies) or iPS-Brew XF (Miltenyi Biotec) media at 37 °C in 5% CO_2_/5% O_2_/90% air humidified atmosphere. Cells were passaged every 4–5 days using StemMACS Passaing Solution (Miltenyi Biotec). For adenovirus transduction experiments, day 2–3 cells were infected for 3 h to overnight and harvested at various time points after infection.

### hiPSC characterization

For flow cytometric analysis of hiPSCs, cells were dissociated into single cells with Accutase Enzyme Cell Detachment Medium (Innovative Cell Technologies) and stained with AlexaFluor 647 anti-human SSEA-4 (Biolegend) and PE mouse anti-human TRA-1-60 (Pharmingen) antibodies. Samples were analyzed using BD Accuri C6 (BD Biosciences) and data were analyzed using FlowJo software (Tree Star, Ashland, OR). For karyotype analysis, iPSCs were cultured in T25 flasks on mouse feeders and live cultures were sent to Cell Line Genetics (Madison, WI) for cytogenetic analysis using G-banded Karyotyping. An average of 20 cells per cell line were analyzed for chromosome integrity.

### Plasmids

Light Switch Promoter Reporter GoClone plasmid with MICU1 promoter sequence cloned (SwitchGear Genomics; S712264) and the corresponding control plasmids were used for luciferase assay. The mutant *micu1*^*Δ3027-297*^ were custom synthesized as gBlock gene fragments from IDT Inc. and subcloned into appropriate vectors for further use. The plasmid was confirmed by sequencing before use.

### RNA interference

hiPSCs (0.5 × 10^6^/well) grown on six-well plates were transfected with pools of 5 distinct siRNAs against Foxd1 (ON-TARGETplus SMARTpool, Dharmacon, USA) (50 nM) using RNAiMAX transfection reagent (Thermo Scientific). As controls, non-targeting siRNA duplexes (Dharmacon) were used.

### Hypoxia/reoxygenation exposure

Freshly isolated NRVMs (rat neonates), hiPSCs, and HPMVEs were subjected to varying hours of hypoxia (5% O_2_–5% CO_2_) followed by reoxygenation (20% O_2_–5%CO_2_) to study the differential expression of MICU1 by hypoxia.

### Western blotting

Cell extracts were prepared using RIPA buffer (50 mM Tris-HCl, pH 7.4, 150 mM NaCl, 0.25% deoxycholic acid, 1 mM EDTA, 1% NP-40, protease inhibitor cocktail (Complete, Roche), and Halt phosphatase inhibitor cocktail (Thermo Scientific). Equal amounts of protein (25 μg/lane) were separated on 4–12% Bis-Tris polyacrylamide gel, transferred to a PVDF membrane using iBlot 2 PVDF regular stacks (Thermo Scientific), and probed with antibodies specific for MCU (1:500, Sigma Aldrich: HPA016480), MICU1 (1:500, in house), MCUR1 (1:500, In house), MICU2 (1:1000, Sigma Aldrich: HPA045511), Actin (1:5000, Santa Cruz: sc-47778), TOM20 (1: 2000, Santa Cruz: sc-17764), cytochrome c (1:5000, Santa Cruz: sc-13156), ATP5A (1:2000, Abcam: ab14748), and Foxd1 (1:500, GeneTex: GTX100271).

### ChIP, quantitative PCR, and luciferase activity

hiPSCs/HPMVECs were grown to ~80 to 90% confluency in a 100-mm culture dish containing the appropriate growth media at normoxic/hypoxic environment. ChIP assay was performed using Pierce^TM^ Magnetic ChIP kit. In brief, DNA-protein complexes were crosslinked and immunoprecipitated using ChIP-validated antibodies against RNA polymerase II, Foxd1 and negative control IgG. Immune complexes were extracted and analyzed by quantitative PCR using primers (Supplementary Table 1) that flank specific regions in the MICU1 promoter. Values were normalized by input DNA. Results were depicted as the fold enrichment over basal expression.

HPMVECs (1 × 10^6^) were transfected with luciferase reporter plasmids (4 μg) containing MICU1 promoter sequence with or without binding elements for Foxd1. After 48 h, cells were exposed to hypoxia (6 h) and reoxygenation (18 h). The cells were lysed, and luciferase activity was measured (LightSwitch Luciferase Assay Reagent) using a plate reader (Infinite M1000 PRO, Tecan).

### Measurement of spontaneous cytoplasmic Ca^2+^ oscillation

Fibroblasts/hiPSCs/cell lineage induced hiPSCs/hiPSCs-CMs with or without MICU1 expression were grown on 0.2% gelatin coated 25 mm glass coverslips. The cells were loaded with 5 μM Fluo-4/AM (30 min) in extracellular medium at room temperature. Coverslips were mounted in an open perfusion microincubator (PDMI-2; Harvard Apparatus) and imaged. Spontaneous Ca^2+^ oscillations were recorded every 3 s (510 Meta; Carl Zeiss, Inc.) at 488 excitations using a ×63 oil objective. Images were analyzed and quantified by using ImageJ (NIH).

### Simultaneous measurement of Ca^2+^ uptake and Δ*Ψ*_m_

An equal number of cells (6 × 10^6^ cells) were washed in Ca^2+^ free PBS, pH 7.4, resuspended and permeabilized with 40 µg/ml digitonin in 1.5 ml of intracellular medium (ICM) composed of 120 mM KCl, 10 mM NaCl, 1 mM KH_2_PO_4_, 20 mM Hepes-Tris, pH 7.2 and 2 µM thapsigargin to block the SERCA pump. All the measurements were performed in the presence of 5 mM succinate. The simultaneous measurement of Δ*Ψ*_m_ and extramitochondrial Ca^2+^ ([Ca^2+^]_out_) clearance as an indicator of _m_Ca^2+^ uptake was achieved by loading the permeabilized cells with JC-1 (800 nM) and Fura2-FF (0.5 µM), respectively. Fluorescence was monitored in a multi-wavelength excitation dual-wavelength emission fluorimeter (Delta RAM, PTI). [Ca^2+^]_out_ is represented as the excitation ratio (340 nm/380 nm) of Fura2-FF/FA fluorescence and Δ*Ψ*_m_ as the ratio of the fluorescence of J-aggregate (570 nm excitation/595 nm emission) and monomer (490 nm excitation/535 nm emission) forms of JC-1. A single Ca^2+^ bolus (1 μM) and mitochondrial uncoupler, CCCP (2 μM), were added at the indicated time points. All the experiments were performed at 37 °C with constant stirring^5,7,11,26^.

### Proteomic analysis

SeV3 cells were infected with Adcontrol andAdMICU1 virus. Cell extracts were prepared using RIPA buffer. The whole cell lysate was identified by label-free proteomics (GeLC-MS/MS)^26^. Mass spectra processing was performed using Proteome Discoverer 1.4.0.288 (DBVersion:79). The generated de-isotoped peak list was submitted to an in-house Mascot server 2.2.07 for searching against the Swiss-Prot database (Release 2013_01, version 56.6, 538849 sequences). Mascot search parameters were set as follows: species, Homo sapiens (Homo sapiens = 20233 sequences); enzyme, trypsin with maximal 2 missed cleavage; fixed modification, cysteine carboxymethylation; variable modification, methionine oxidation; 10 ppm Da mass tolerance for precursor peptide ions; and 0.2 Da tolerance for MS/MS fragment ions. All peptide matches were filtered using an ion score cutoff of 20. Following identifications, MS spectra were uploaded to the LC-MS ProGenesis 4.1 software to generate list of proteins that are differentially expressed in SeV3 with ectopic expression of MICU1.

### Measurement of mitochondrial superoxide

Mitochondrial superoxide was measured by using the mitochondrial oxygen free radical indicator MitoSOX Red (molecular probes; Invitrogen). Briefly, cells grown on 0.2% gelatin coated glass coverslips were loaded with 5 μM MitoSOX Red for 30 min at 37 °C, and coverslips were mounted in an open perfusion microincubator (PDMI-2; Harvard Apparatus) and imaged. Confocal (510 Meta; Carl Zeiss, Inc.) images were obtained at 561 nm excitation by using a ×63 oil objective. Images were analyzed, and the mean MitoSOX Red fluorescence was quantified by using Image J software (NIH).

### Mitochondrial oxygen consumption rate

Intact SeV3-CMs/NRVMs were subjected to oxygen consumption rate (OCR) measurement at 37 °C in an XF96 extracellular flux analyzer (Seahorse Bioscience). SeV3-CMs/NRVMs (3 × 10^5^) were sequentially challenged with oligomycin, FCCP, and rotenone plus antimycin A for measurement of OCR^69^. For measurement of glucose or fatty acid oxidation glucose or carnitine were used as substrates respectively.

### Induction of lineage-specific differentiation of hiPSCs

Exponentially growing SV20 hiPSCs were infected with MICU1 adenovirus or control virus overnight. Cells were harvested 2 days later and replated on Geltrex-coated 24-well plates at 1 × 10^5^ cells/well (ectoderm) and 4 × 10^5^ cells/well (endoderm and mesoderm) and cultured overnight. STEMdiff^TM^ Trilineage Differentiation Kit (Stem Cell Technologies) was used to induce specification of cells of the three germ layers. Differentiating cells were assayed at various time points of the differentiation time course (5 days for endoderm and mesoderm; 7 days for ectoderm).

### Quantitative measurement of lineage-specific markers

Differentiating hiPSCs were harvested and total RNA was isolated using a PureLink RNA mini kit (Thermofisher Scientific). cDNA was generated using a High Capacity cDNA Reverse Transcription Kit (Applied Biosystems). Semi-quantitative real-time PCR was performed using SYBR green reagents and primers against lineage-specific marker genes (Supplementary Table 1).

### Generation of hiPSCs-derived cardiomyocytes (iPSCs-CMs)

hiPSC-cardiomyocytes (iPSCs-CMs) were generated using standard protocols^55,56^. In general, cells used were >95% positive for cTnT staining by FACS. Differentiated cells were re-plated at 6 × 10^5^ cells/well in 6-well plates for calcium transients measurement and 2 × 10^4^ cells/well for Seahorse experiments and maintained in RPMI media plus B27 Supplement (cat#17504044, Thermofisher Scientific) before they were analyzed. Differentiated iPSCs-CMs were harvested and total RNA was isolated using a PureLink RNA mini kit (Thermofisher Scientific). cDNA was generated using a High Capacity cDNA Reverse Transcription Kit (Applied Biosystems). Semi-quantitative real-time PCR was performed using SYBR green reagents and primers against Mef2C, MYH7, and MYH6 (Supplementary Table 1).

### Quantification and statistical analysis

Data from multiple experiments were quantified and expressed as mean ± SEM., and differences between groups were analyzed by using two-tailed paired Student’s *t*-test. Differences in the means among multiple data sets were analyzed using 1-way ANOVA with the Kruskal–Wallis test, followed by pairwise comparison using the Dunn test. *P* value <0.05 was considered significant in all analysis. The data were computed with either Graphpad Prism version 7.0 or SigmaPlot 11.0 Software.

## Electronic supplementary material

Supplementary Information

Peer Review

## Data Availability

The data that support the findings of this study are available from the corresponding author upon reasonable request. The proteomics data that support the findings of this study are available from peptideatlas, with the accession code PASS01222.
