# Supplementary material for: FOXD1-dependent MICU1 expression regulates mitochondrial activity and cell differentiation
Source: Nat Commun. 2018 Aug 29;9:3449. doi: 10.1038/s41467-018-05856-4 (PMC6115453; doi:10.1038/s41467-018-05856-4)
Supplement: Supplementary file 1 — Supplementary Information [file 41467_2018_5856_MOESM1_ESM.pdf]

Supplementary information

FOXD1-dependent MICU1 expression regulates mitochondrial activity and cell differentiation

Shanmughapriya et al.

SUPPLEMENTARY INFORMATION

The supplemental data includes five Supplementary figures with figure legends and one supplementary table.

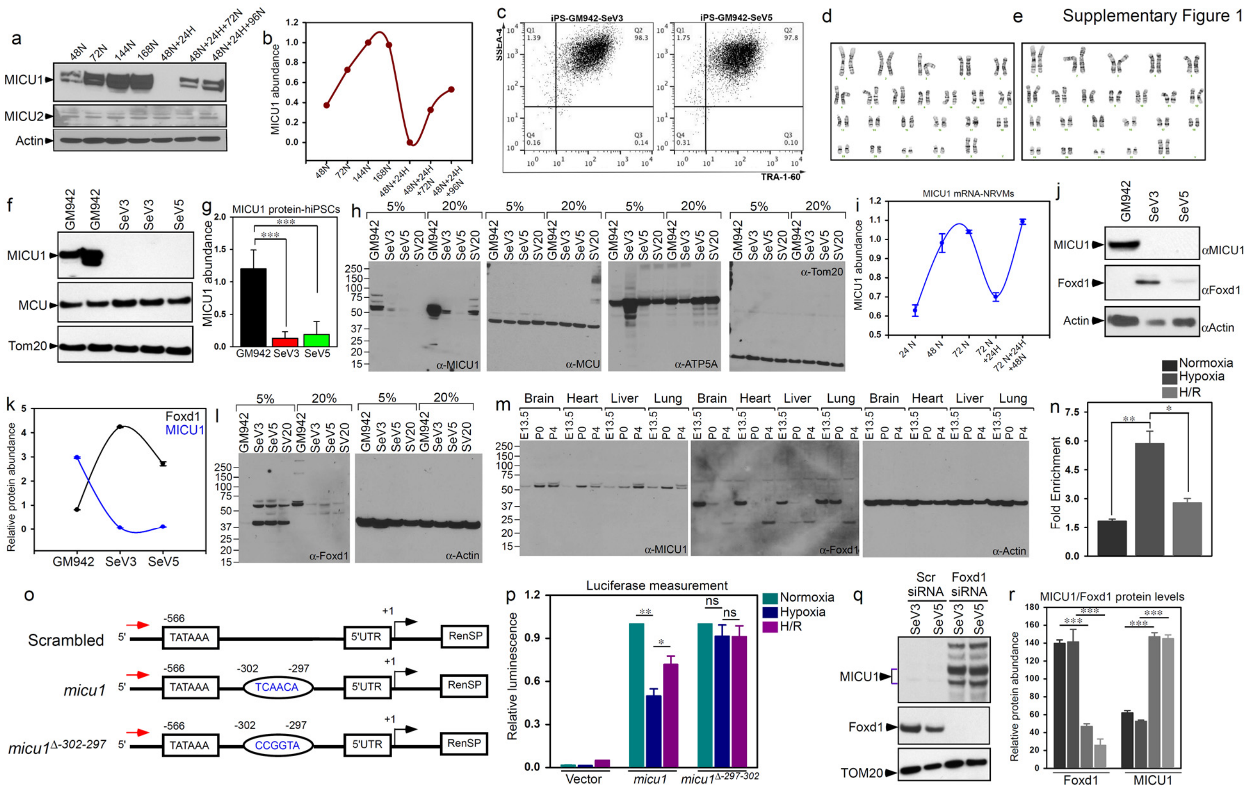

Supplementary Figure 1. MICU1 is differentially regulated by hypoxia. (**a**) Representative Western blot for lysates from NRVMs exposed to normoxia/hypoxia and probed with antibody specific for MICU1, MICU2, and actin. (**b**) Quantification of relative MICU1 abundance from (a). (**c**) Quantification of TRA-1-60 and SSEA-4 protein expression by FACS for iPS-GM942-SeV3 and iPS-GM942-SeV5. (**d** and **e**) SeV3 and SeV5 iPSC lines display a normal karyotype by G-banding analysis. (**f**) Representative Western blot for lysates from control fibroblasts (GM942), hiPSCs (SeV3 and SeV5) probed with antibody specific for MCU, MICU1, and Tom20. (**g**) Quantification of relative MICU1 protein abundance from (f). (**h**) Representative Western blot for lysates from control fibroblasts and hiPSCs grown under hypoxic/normoxic conditions and probed with antibody specific for MICU1, MCU, ATP5A, and Tom20. (**i**) Quantification of MICU1 mRNA levels in NRVMs exposed to hypoxia or normoxia. (**j**) Representative Western blot for lysates from control fibroblasts and hiPSCs probed with antibody specific for MICU1, Foxd1, and actin. (**k**) Quantification of relative MICU1 and Foxd1 abundance from (j). (**l**) Representative Western blot for lysates from control fibroblasts and hiPSCs grown under normoxic/ hypoxic conditions and probed with antibody specific for Foxd1 and β-actin. (**m**) Representative Western blot for lysates from brain, heart, liver, and lung harvested from embryos/neonates and probed with antibodies specific for MICU1, Foxd1, and β-actin. (**n**) ChIP-assay was performed in

HPMVECs exposed to normoxia and hypoxia. Antibody specific for Foxd1 was used to immunoprecipitate the chromatin and the fold enrichment of *micu1* promoter relative to the matched input control was quantified by qRT-PCR. (**o**) Schematic of the *micu1* promoter-luciferase constructs. Foxd1 consensus response elements are shown in ovals. (**p**) HPMVECs transfected with *micu1* promoter-luciferase constructs were exposed to normoxia and hypoxia and analyzed for luciferase activity. (**q**) Representative Western blot for lysates from control and Foxd1 KD hiPSCs probed with antibody specific for Foxd1, MICU1, and Tom20. (**r**) Quantification of relative protein abundance of Foxd1 and MICU1. Data are Mean ± SEM, n=3-5. Data represents Mean ± SEM; *P < 0.05, **P < 0.01, ***P <0.001 n = 3-5.

Supplementary Figure 2

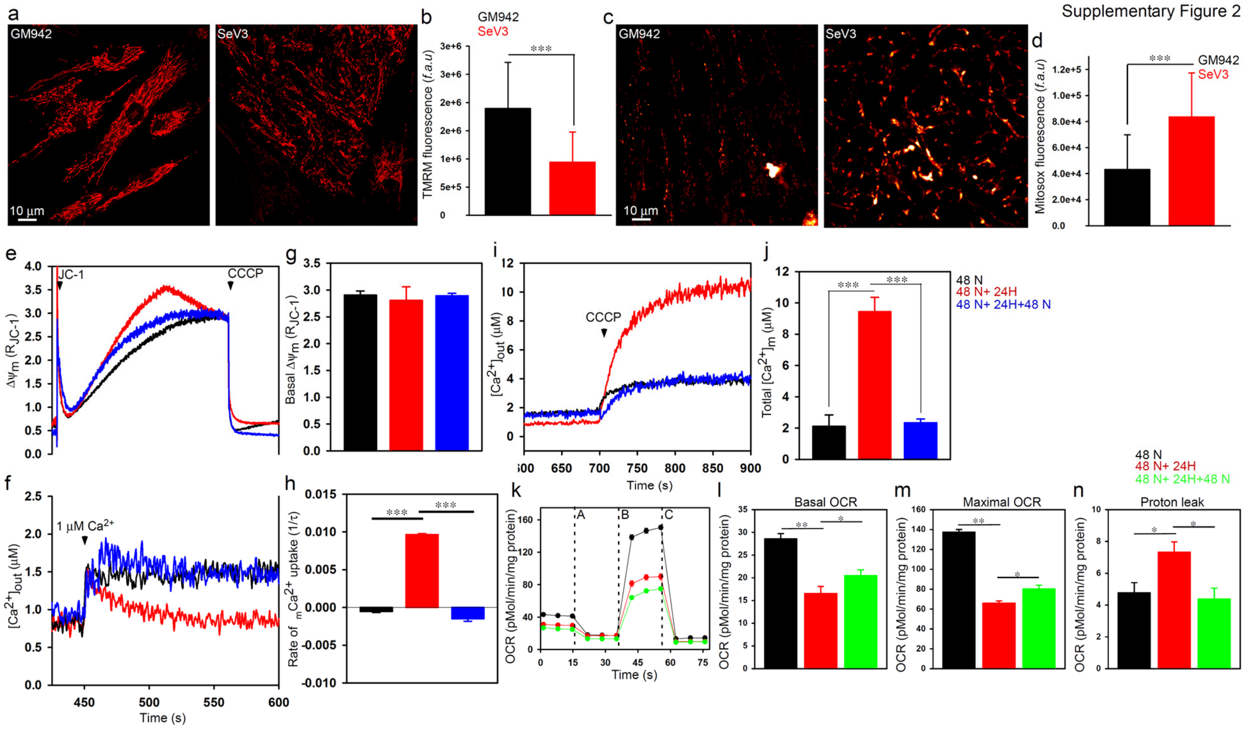

Supplementary Figure 2. Loss of MICU1 in hiPSCs results in mitochondrial matrix Ca^{2+} overload and mROS generation. (**a**) Representative confocal images of control fibroblasts and hiPSCs stained with TMRM, a $\Delta\Psi m$ indicator. (**b**) Quantification of TMRM fluorescence in GM942, and SeV3. Data indicate Mean ± SEM; ***P < 0.001; n = 3-4. (**c**) Representative confocal images of control fibroblasts and hiPSCs stained with MitoSox, a mitochondrial ROS indicator. (**d**) Quantification of MitoSox fluorescence in GM942, and SeV3. Data indicate Mean ± SEM; ***P < 0.001; n = 3-4. (**e**) Mean traces of $\Delta\psi_m$ in permeabilized NRVMs exposed to hypoxia/reoxygenation. (**f**) Mean traces of $[Ca^{2+}]_{out}$ measured in permeabilized NRVMs exposed to hypoxia/reoxygenation. (**g**) Quantification of basal $\Delta\psi_m$ before addition of CCCP. Data represents Mean ± SEM; n = 4-6. (**h**) Quantification of the rate of $_mCa^{2+}$ uptake as a function of decrease in bath Ca^{2+} fluorescence after an extramitochondrial Ca^{2+} pulse (1 µM). Data represents Mean ± SEM; ***P <0.001; n = 4-6. (**i**) Mean traces of $[Ca^{2+}]_{out}$ after addition of CCCP. (**j**) Quantification of matrix Ca^{2+} after the addition of CCCP. Data represents Mean ± SEM; ***P <0.001; n = 4-6. (**k**) Measurement of oxygen consumption rate (OCR) in NRVMs exposed to hypoxia and reoxygenation. After basal OCR, oligomycin (A), FCCP (B), and rotenone + Antimycin A (C) were added as indicated. (**l-n**) Bar represents mean basal OCR (**l**), maximal OCR (**m**), and proton leak (**n**). Data indicate Mean ± SEM; *P<0.05, ** P <0.01; n=4.

Supplementary Figure 3

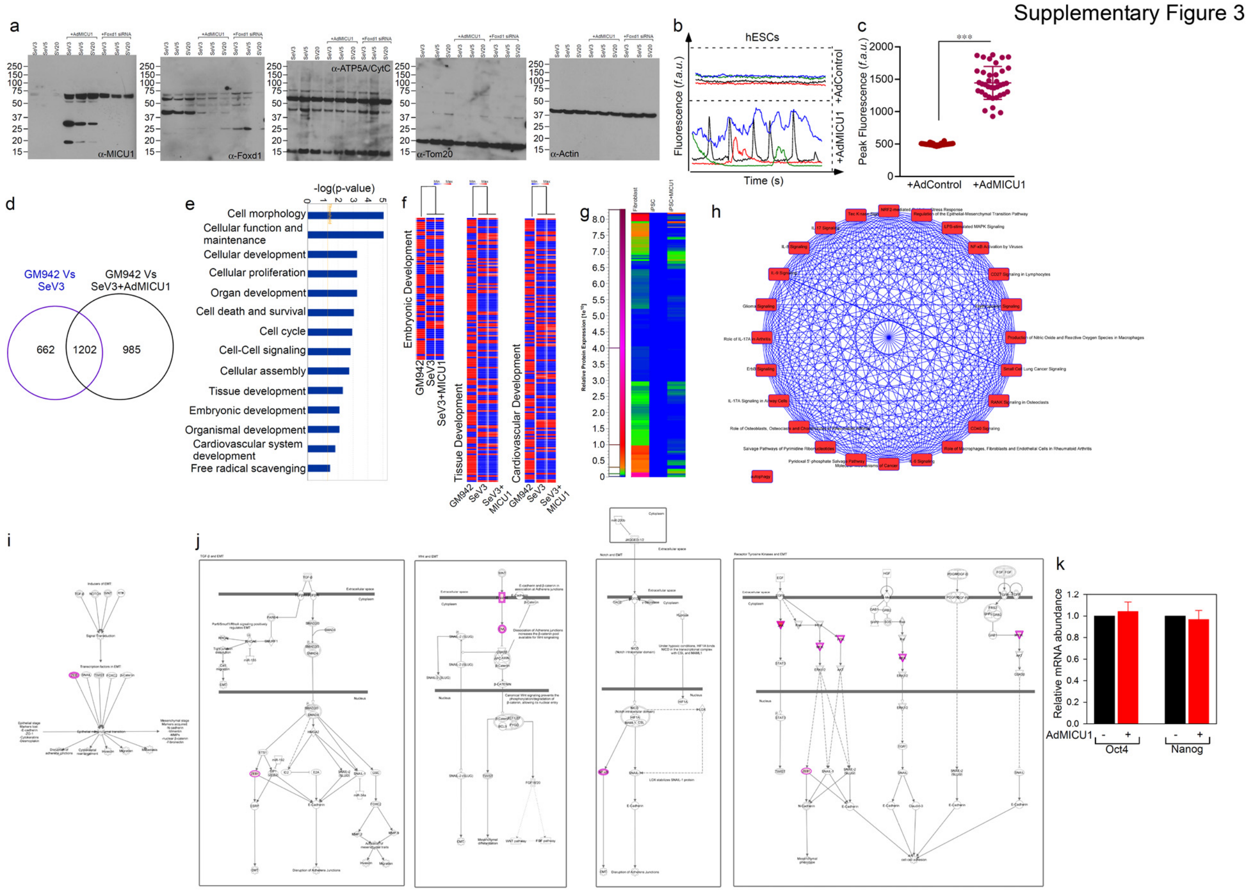

Supplementary Figure 3. Ectopic expression of MICU1 in hiPSCs facilitates EMT.
(**a**) Representative Western blot for lysates from hiPSCs with and without MICU1 expression. The Western blots were probed with antibody specific for MICU1, MCU, ATP5A, Cytochrome C, Foxd1, Tom20, and β-actin. (**b**) Traces of spontaneous cytosolic Ca^{2+} oscillations in hESCs with and without MICU1 expression. Intact cells were loaded with fluo4 and the spontaneous Ca^{2+} oscillations were observed at 488 nm. (**c**) Quantification of peak fluo-4 fluorescence. Data indicate quantified individual peak fluorescence. ***$P < 0.001$; n = 3-4. (**d**) Overlap and correlation between protein expressions in GM942 compared with SeV3 and SeV3+MICU1. (**e**) Functional enrichment map for protein MS-based expression revealing several pathways modulated by MICU1 expression in hiPSCs. (**f and g**) Clustered heat map to depict differential expression of proteins. (**h**) Functional enrichment map for protein MS-based expression revealing several pathways modulated by MICU1 expression in hiPSCs. Only the subset of pathways that have significant overlap with cellular differentiation and development is shown. (**i and j**) The EMT pathway that is modulated by MICU1 expression is depicted. The EMT pathway is represented in each column and the proteins that significantly depend on Ca^{2+} is highlighted.

(**k**) Quantification of mRNA abundance of the pluripotency markers, Oct4, and Nanog in hiPSCs with or without MICU1 expression.

Supplementary Figure 4

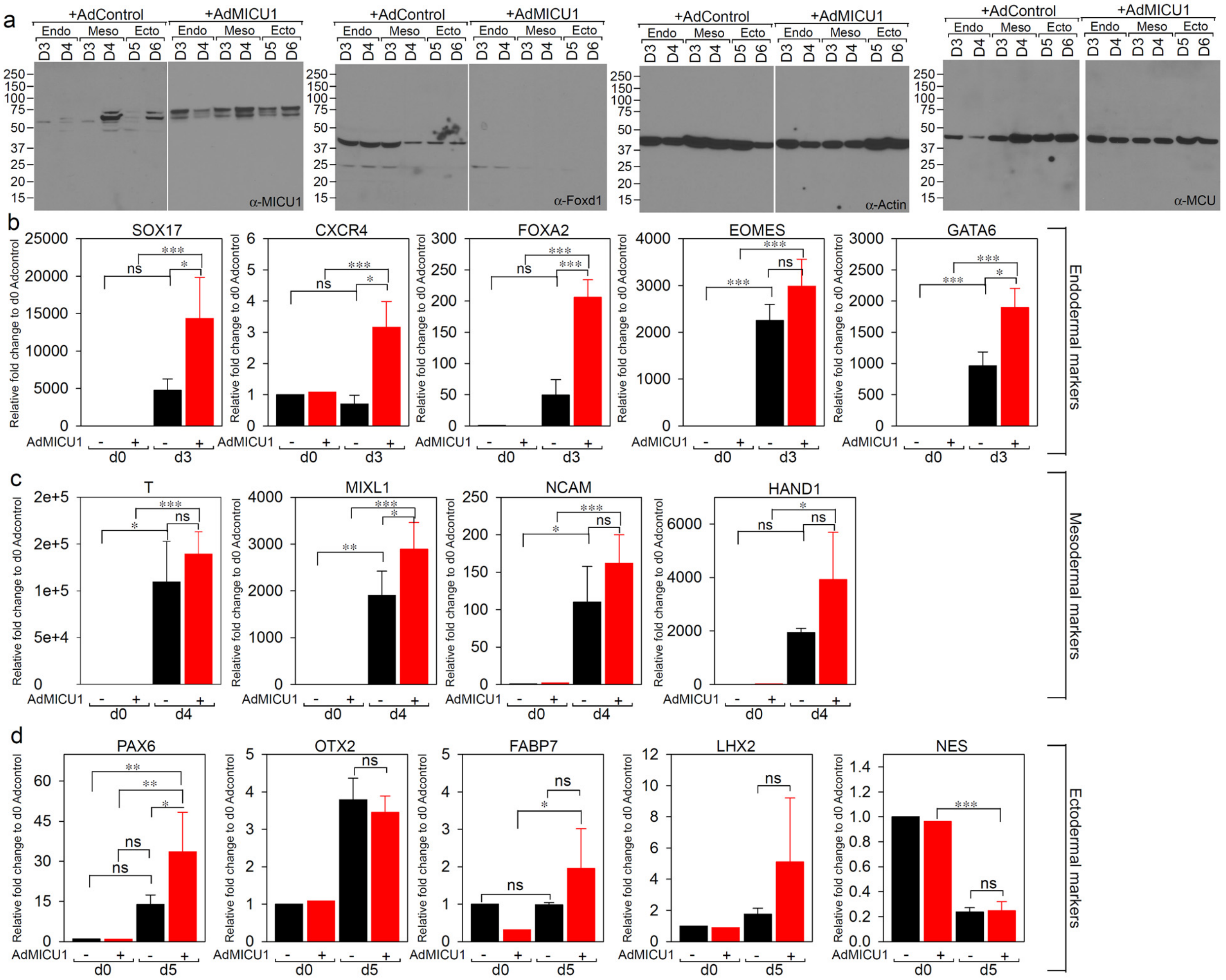

Supplementary Figure 4. Ectopic expression of MICU1 in hiPSCs modulates cell differentiation. (**a**) Representative Western blot for lysates from hiPSCs induced for lineage specific differentiation for the specified days with and without MICU1 expression. The Western blots were probed with antibody specific for MICU1, MCU, Foxd1, and β-actin. (**b**) Quantification of mRNA abundance of the endodermal markers, SOX17, CXCR4, FOXA2, EOMES, and GATA6 in hiPSCs induced for endoderm specific differentiation at day 3. (**c**) Quantification of mRNA abundance of the mesodermal markers, brachyury (T), MIXL1, NCAM, and HAND1 in hiPSCs induced for mesoderm specific differentiation at day 4. (**d**) Quantification of mRNA abundance of the ectodermal markers, PAX6, OTX2, FABP7, LHX2, and NES in hiPSCs induced for ectoderm specific differentiation at day 5. Data indicate Mean ± SEM; n = 3-4. *P < 0.05, **P < 0.001, ***P < 0.0001; n = 3-4.

Supplementary Figure 5

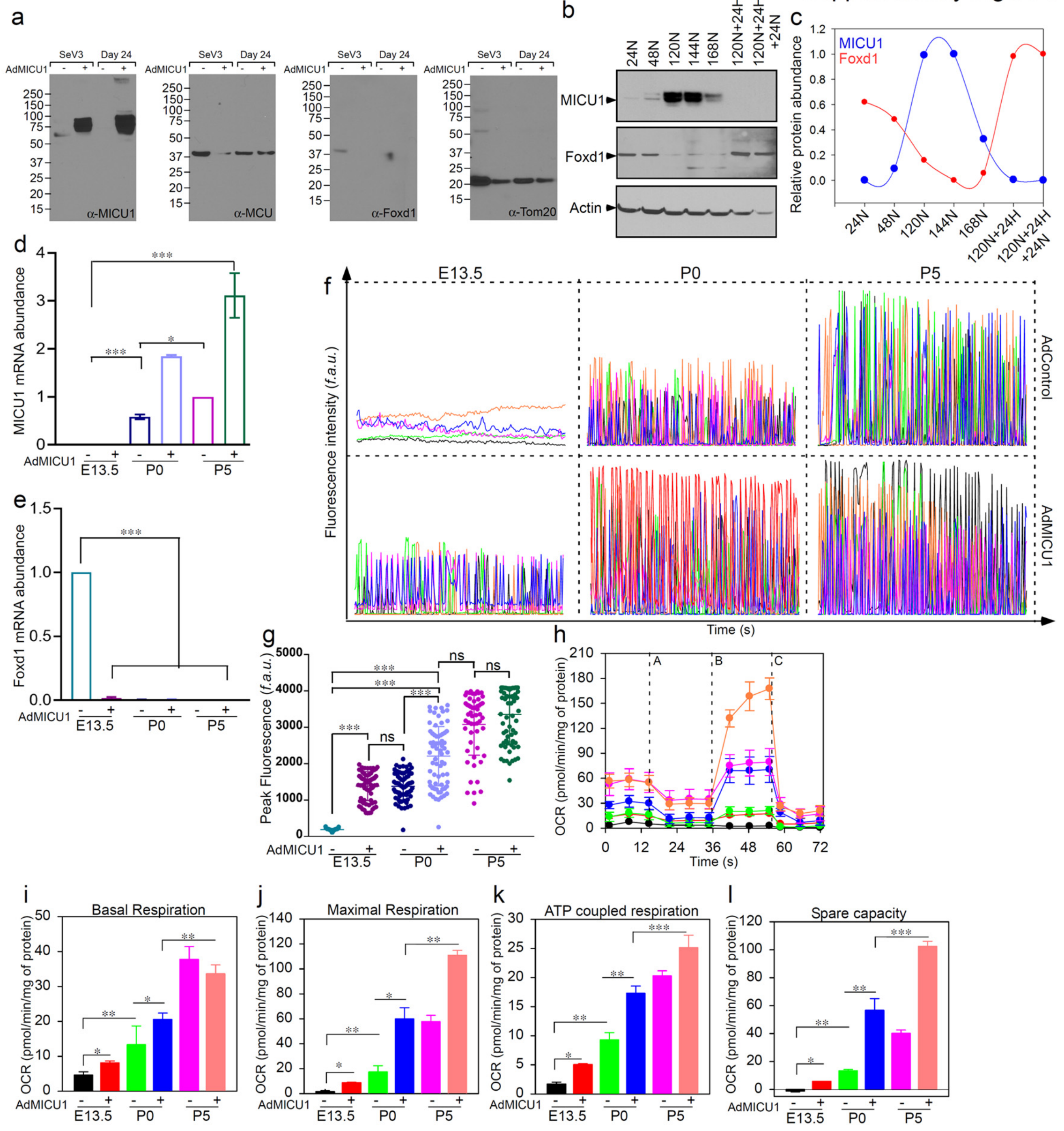

Supplementary Figure 5. MICU1 expression promotes myocyte maturation. (**a**) Representative Western blot for lysates from SeV3 and SeV3-derived cardiomyocytes (SeV3-CMs) with and without MICU1 expression. The Western blots were probed with antibody specific for MICU1, MCU, Foxd1, Tom20, and β-actin. (**b**) Representative Western blot for lysates from NRVMs exposed to hypoxic or normoxic conditions. The Western blots were probed with antibody specific for MICU1, Foxd1, β-actin. (**c**) Quantification of the MICU1 and Foxd1 protein abundance in NRVMs as quantified from (a). (**d and e**) Quantification of the MICU1 (c) and Foxd1 (d) mRNA abundance in myocytes isolated from embryos or neonates. (**f**)

Traces of spontaneous cytosolic Ca^{2+} oscillations in myocytes isolated from embryos or neonates with and without MICU1 expression. (**g**) Quantification of peak fluo-4 fluorescence. Data indicate quantified individual peak fluorescence. *P<0.05, ***P < 0.001; n = 3-4. (**h**) Measurement of oxygen consumption rate (OCR) in myocytes isolated from embryos or neonates with or without MICU1 expression using carnitine as substrate. After basal OCR measurement, oligomycin (A), FCCP (B), and rotenone + Antimycin A (C) were added as indicated. Representative traces of OCR in myocytes isolated from embryos or neonates are shown. (**i-l**) Quantification of basal (h), maximal (i), ATP coupled respiration (j), and spare capacity (k) in myocytes isolated from embryos or neonates. Data indicate Mean ± SEM; *P < 0.05, **P<0.01, ***P<0.0001; n = 3-4.

Supplementary Table 1: List of primers used in the study

Primer name	Forward sequence	Reverse sequence
**Human Primers**		
TBP	TGAGTTGCTCATACCGTGCTGCTA	CCCTCAAACCAACTTGTCAACAGC
SOX17	CTCTGCCTCCTCCACGAA	CAGAATCCAGACCTGCACAA
CXCR4	CCCTCCTGCTGACTATTCCC	TAAGGCCAACCATGATGTGC
FOXA2	GCATTCCCAATCTTGACACGGTGA	GCCCTTGCAGCCAGAATACACATT
EOMES	AGCTCTCCAAGGAGAAAGTG	GCCTTCGCTTACAAGCACTG
GATA6	TCTACAGCAAGATGAACGGCCTCA	TCTGCGCCATAAGGTGGTAGTTGT
BRACHURY (T)	TGTCCCAGGTGGCTTACAGATGAA	GGTGTGCCAAAGTTGCCAATACAC
MIXL1	ACGTCTTTCAGCGCCGAACAG	TTGGTTCGGGCAGGCAGTTCA
NCAM	ATGGAAACTCTATTAAAGTGAACCTG	TAGACCTCATACTCAGCATTCCAGT
HAND1	TCAAAGACGCACTCTTCCAC	GTGCAGCGACAAAAAGAAAA
PAX6	CTGAAGCGGAAGCTGCAAAG	TTGCTGGCCTGTCTTCTCTG
OTX2	GACCACTTCGGGTATGGACT	TGGACAAGGGATCTGACAGT
FABP7	TGTGACCAAACCAACGGTAAT	CTTTGCCATCCCATTTCTGTA
LHX2	GAAGGGGCGGCCGAGGAAAC	GCTGGTCACGGTCCAGGTGC
NES	GCGTTGGAACAGAGGTTGGA	TGGGAGCAAAGATCCAAGAC
MEF2C	GACCTCACGTCTGGTGCAG	TGCTTGCATATTCTTGTTCAAGTT
MYH6	TCAGCTGGAGGCCAAAGTAAAGGA	TTCTTGAGCTCTGAGCACTCGTCT
MYH7	TCGTGCCTGATGACAAACAGGAGT	ATACTCGGTCTCGGCAGTGACTTT
MICU1	CTTCCTCGAATTTCAGCGTAAAC	CACCAAACTGCCTCTCAGTAA
OCT4	AACCTGGAGTTTGTGCCAGGGTTT	TGAACTTCACCTTCCCTCCAACCA
NANOG	CCAACATCCTGAACCTCAGC	GCTATTCTTCGGCCAGTTG
MCU	CAGTTCACACTCAAGCCTATCT	ATCAAGGAGGAGGAGGTCTATT
Foxd1	TATGACCCTGAGCACTGAGAT	CCTCCTCTCCTCGTCTTCTT
MICU2	GTCAGCAGGAGAGAGCATTAG	GCTCTCTTAAACTCCGCTAGTC
**Mouse Primers**		
MCU	GACCTCCTAAGCCATGAAGATG	AGCTCCCGCTCTTTGTTAAG
MICU1	AATTGCCCAGGAACGAGAAA	GAGGACTGTTGTGAGGAAGATG
Foxd1	GCTAAGAATCCGCTGGTG AA	CTGCTGATGAACTCGCAGAT
**CHIP primers**		
MICU1 promoter	GTCTCCGCTGTTTCATCTCTA	TCTCCTTATACCCTCCAGAGTC
