# Supplementary material for: FOXD1-dependent MICU1 expression regulates mitochondrial activity and cell differentiation
Source: Nat Commun. 2018 Aug 29;9:3449. doi: 10.1038/s41467-018-05856-4 (PMC6115453; doi:10.1038/s41467-018-05856-4)
Supplement: Supplementary file 2 — Peer Review [file 41467_2018_5856_MOESM2_ESM.pdf]

Reviewers' comments:

Reviewer #1 (Remarks to the Author):

The paper submitted by Shanmughapriya et al investigates the regulation of the MCU complex during development. The authors show that the MCU gatekeeper MICU1 undergoes to transcriptional regulation during embryo maturation, an event controlled by O2 levels through the Foxd1 repressor. Overall, the findings are novel, interesting and experiments well performed, as one would expect from a leading lab in the field. However, some conclusions require additional evidences:
• In the introduction, the authors state "hypoxia increases ROS via transfer of electrons from ubisemiquinone to O2 at the Q0 site of complex III of the mitochondrial ETC". Although some data support this notion, I think the is no general consensus on this point. The idea that ROS are generated when the key substrate (O2) is absent (i.e. during hypoxia) is counterintuitive. If the authors want to support this notion, they should measure ROS in the different experimental conditions. Otherwise, they should reword that sentence
• According to the authors, MICU1 is regulated at transcriptional level. However, mRNA is measured only hypoxic condition (Fig. 2a) but not during development (fig 1). I think this is a critical point, since the underlying hypothesis is that developmental changes are mimicked by hypoxia. However, the author should provide direct evidences that MICU1 is regulated at transcriptional level during development (i.e. in all conditions tested in Fig.1). And what about Foxd1 levels during development?
• In Fig 1l, MICU1 levels are missing in SeV5 line
• Figure 2b lacks loading controls
• I can't understand why the authors used a different cellular model (HPMVEC) for ChIP. This must be better discussed.
• Panel 2f: in this experimental setup, it looks like there is still a substantial MICU1 expression during hypoxia. Why?
• Panel 2g: why so many MICU1 bands are present when Foxd1 is silenced?
• Fig 3a and b show that mitochondrial membrane potential is decreased in SeV3 cells. According to the authors, this is a consequence of MICU1 levels. However, MICU1 downregulation has no effect of organelle membrane potential in other cellular models. Of course, a number of other reasons could account for this difference, and I don't think MICU1 levels are sufficient to explain changes in membrane potential.
• Given the impact of MICU1 expression on gene expression (fig 4), I think it would be worth to test the differentiation potential of these cells into different lineages

Minor points:
• Page 1: in the introduction paragraph, the reference to the identification of MCU by the Rizzuto group is lacking (refs 8-10)
• In some panels, expression levels are shown as smoothed traces (1j, 2a, 2c, 2j and 5b), and I think this is misleading. Bar graphs should be used instead, as in the other panels.

Reviewer #2 (Remarks to the Author):

In this study, Shanmughapriya and colleagues report the novel finding that MICU1 expression is regulated by hypoxia in a FOXD1-dependent manner.

They also provide evidence that MICU1 is constitutively down-regulated in IPSCs. Furthermore, as previously reported they show that MICU1 functions as a gatekeeper of mitochondrial Ca++ since in absence of MICU1 mitochondrial Ca++ accumulates and this is associated to increased ROS.

Lastly, they claim that cell differentiation is somehow associated to decreased levels of MICU1.

The paper is potentially interesting. However, at this stage it is a descriptive and confusing mosaic of data with no clear take home message. The authors' conclusions are not fully supported by the data as shown. The most solid findings are the ones that confirm previous reports.

Specific Comments:
- The authors use at least three different cell types (NRVM, HPMVECs and IPSCs), and throughout the paper they keep switching between cell types without providing any rationale but simply increasing the level of confusion and misleading statements.
- It is not at all clear why increased Ca++ levels in the mitochondria augments ROS and reduce OCR, at least in hypoxia.
- It is completely elusive why hypoxia would cause uncoupling of the oxidative phosphorylation.
- More importantly, there is no solid and convincing evidence that decreased levels of MICU1 impair cell differentiation, which in principle would be the most novel aspect of the paper.
- The title is an overstatement.

Reviewer #3 (Remarks to the Author):

In this manuscript, Shanmughapriya and co-workers investigate expression and function of Mitochondrial Ca2+ uptake protein 1 (MICU1) in several experimental cell systems. Respective systems include human fibroblasts and hiPSCs (derived from the corresponding fibroblasts), human pulmonary microvascular endothelial cells (HPMVECs), neonatal rat ventricular myocytes (NRVMs), rodent ventricular myocytes from several embryonic/post-natal stages; gene expression in rodent tissues throughout development was investigated as well.
Specific parts of the manuscript in particular investigations on the interplay of FOXD1 and MICU1 expression and its consequences of Ca2+ ion oscillation in human fibroblast versus hiPSC are interesting. Unfortunately, key data in this part of the paper remain elusive. Other parts are poorly integrated into the context and authors make irrelevant claims based on very poor data sets.

Specific issue:
Abstract / Introduction: overall structure and content is difficult to read and understand. E.g. odd list and order in the first sentence: "The morphological changes occurring between early and late embryonic stages coincide with several factors (mechanical load, contractile force generation, blood flow, mechanical stress, and hemodynamics)… " What are the specific differences between: mechanical load versus mechanical stress ?, blood flow versus hemodynamics?
Moreover: "The morphological changes occurring between early and late embryonic stages …. facilitate cellular differentiation. Despite these factors, the role of mitochondria in cell morphogenesis during development remains unexplored." A meaningful connection between these sentences remains elusive.
Figures 1 / 2: GM942 fibroblast and iPS-SeV3 and iPS-SeV5 (resulting from reprograming of these fibroblast) are analyzed if Figures 1k-i and in Figures 2b-c and onwards. Surprisingly, MICU1 mRNA abundance in response of to normoxia/hypoxia treatment, which is presented in the "intercalated" Figure 2a, is not tested in fibroblasts/ iPS but analyzed in primary cardiomyocytes (NRVMs). Notably, the unexpected (and inappropriate) switch in cell systems is not even mentioned in the main text but only hidden in figure legends. This is strange and the expression of MICU1 (on the RNA and protein level) in response to normoxia/hypoxia must be investigated in GM942 and iPS-SeV3/ iPS-SeV5, respectively to make the study conclusive.
Furthermore, authors are presenting 1) NRVMs data in Fig. 2a, 2) GM942 and iPS-SeV3/ iPS-SeV5 data in Fig. 2b-c, and 3) HPMVECs for ChIP-analysis and other assays in Fig 2d-j. This heterogeneous intermingling of different cell systems and data is confusing and not well justified in the manuscript.

Figure 4 a, b: The induction Ca2+ oscillation in hiPSC by MICU1 overexpression is the most interesting part of the paper. However, if there is such direct relation between FOXD1> (inhibiting MICU1) MICU1 > (inducing Ca2+ oscillation) and Ca2+ oscillation as authors' suggest, it's expected that FOXD1 knockdown by siRNA (Figure 2 g, h) should also re-establish Ca2+ oscillation in hiPSC equivalent to MICU1 gain of function in hiPSC (Figure 4 a, b); this must be demonstrated.

Authors found that: "Also, an epithelial to mesenchymal transition (EMT) pathway proteins, N-Cadherin, Vimentin, MMPs, nuclear β-catenin, and fibronectin were enriched in hiPSCs expressing MICU1 (Supplementary Figure 2b-2d)." If these pathways are substantially modulated by MICU1 expression in hiPSC, it deems surprising that maintenance of the pluripotent phenotype was not disrupted. Therefore, after reconstitution of MICU1 expression in hiPSC, the following assays must be tested and shown: conventional flow cytometry analysis for pluripotency markers (TRA1-60, 1-80, SSEA1,3,4, Nanog, Oct3/4), testing hiPSC proliferation kinetics (compared to untreated controls) immunofluorescence staining of hiPSC colonies. Please also indicate: can these cells being maintained / passaged long term? It's also not clear from the paper: was FOXD1 expression in hiPSC monitored in response to MICU1 overexpression?

Authors make the following claim: "Reconstitution of MICU1 either by Foxd1 knock down or ectopic expression restores the periodic cytosolic Ca2+ oscillations that is a prerequisite for

cellular differentiation." If this hypothesis holds true, it's expected that Ca2+ oscillations in human pluripotent stem cells (hPSC) is induced early during differentiation. To confirm their data / hypothesis the following is required: 1) monitoring the lack of Ca2+ oscillation at the pluripotent state in additional hiPSC and hESC lines, 2) induction of Ca2+ oscillation should be observable shortly upon induction of undirected (e.g. via EB formation in FCS media) or lineage specific differentiation (e.g. using published protocols for Endo,- Meso-, and Ecto-dermal derivatives) of hiPSC.

Figure 5 A: The immunofluorescence pictures comparing primary CMs with / without MICU1 overexpression are subjective and non-informative; relevant conclusion on sarcomere structures cannot be made from these pictures.
Figure 5B: Quantifying the fluorescence intensity and showing it via an "oscillation patterns" is nonsense with respect to the applied assay ology.
Authors: "Expression of MICU1 (AdMICU1) in E9.5, and P0 caused a significant increase in the number of Z-bands compared to E9.5 and P0 WT controls (Figure 5a and b)." It remains elusive which assays have been applied to support this claim. Overall, this is the weakest part of the manuscript. It is poorly interlinked with other parts of the paper and should be removed as it is far-fetched and claims are not supported by relevant data.

At the end of the paper it is stated that: "We establish that the differential expression of MICU1 is essential in maintaining developmental plasticity, thereby facilitating adaptation to stress conditions and to allow a well-integrated tissue repair response." In view of this reviewer, this is a massive overstatement based on the data basis.

Responses to reviewers' comments

We would like to thank the reviewers for their constructive comments and suggestions. Below we detail the changes that we have undertaken in the revised manuscript that address the reviewers' queries. To establish the transcriptional regulation of MICU1 during development, we conducted most of the suggested experiments that further strengthen the manuscript. To avoid confusion to the best of our possibility we have tried to use single cellular model and the use of other cellular models were clearly justified in the revised manuscript.

Reviewer #1

The paper submitted by Shanmughapriya et al investigates the regulation of the MCU complex during development. The authors show that the MCU gatekeeper MICU1 undergoes to transcriptional regulation during embryo maturation, an event controlled by O2 levels through the Foxd1 repressor. Overall, the findings are novel, interesting and experiments well performed, as one would expect from a leading lab in the field. However, some conclusions require additional evidences:

We thank the reviewer for their invaluable time and constructive comments.

1. In the introduction, the authors state "hypoxia increases ROS via transfer of electrons from ubisemiquinone to O2 at the Q0 site of complex III of the mitochondrial ETC". Although some data support this notion, I think the is no general consensus on this point. The idea that ROS are generated when the key substrate (O2) is absent (i.e. during hypoxia) is counterintuitive. If the authors want to support this notion, they should measure ROS in the different experimental conditions. Otherwise, they should reword that sentence

Response: During hypoxia, lack of O_2 largely inhibits the electron transport chain, and therefore, ATP synthesis is blocked. During this process, $\Delta\Psi_m$ is maintained by the reverse mode of ATP synthase, hydrolyzing remaining ATP and translocating H+ into the intermembrane space. **Supplementary Fig. 2n** represents an increase in the proton leak with a concomitant increase in electron leak that reacts with existing molecular oxygen thus generating mROS.

2. According to the authors, MICU1 is regulated at transcriptional level. However, mRNA is measured only hypoxic condition (Fig. 2a) but not during development (fig 1). I think this is a critical point, since the underlying hypothesis is that developmental changes are mimicked by hypoxia. However, the author should provide direct evidences that MICU1 is regulated at transcriptional level during development (i.e. in all conditions tested in Fig.1). And what about Foxd1 levels during development?

Response: We thank the reviewer for this thoughtful comment. Though we showed Foxd1 to bind the *micu1* promoter by Chip assay and the binding of Foxd1 to depend on oxygen deprived condition, we did not provide the mRNA levels of MICU1 and Foxd1 in the original submission. Taking reviewer's concern into consideration we have now assessed the MICU1 mRNA levels in tissue samples harvested from different embryonic development stages by qRT PCR and data is included in **Fig. 1k**. To further substantiate the role of Foxd1 to regulate MICU1 expression, Foxd1 mRNA and protein levels were measured by qRT PCR and Western blot analysis respectively in tissue samples and new data incorporated as **Fig. 2h-2j**. Our new results strengthen our hypothesis of an inverse regulation of MICU1 and Foxd1 levels during developmental stages. Also, the inverse regulation of MICU1 and Foxd1 as seen in **Fig. 1k, 2b, 2f, 2g, 2h, 2i, 2j, 4a, Supplementary Fig. 1i and 1j, and Supplementary Fig. 5a and 5b** reinforce Foxd1-mediated transcriptional regulation of MICU1 during hypoxia.

3. In Fig 1I, MICU1 levels are missing in SeV5 line

Response: Although we have shown the protein levels of MICU1 in SeV5 lines, we now quantified the MICU1 abundance in SeV5 and the quantification is incorporated in the revised manuscript as **Supplementary Fig. 1g**. Additionally, MICU1 abundance was measured in additional hiPSCs/hESCs: two clonal hiPSC lines (SeV3 and SeV5) generated from skin fibroblasts of one individual, one hiPSC line (SV20) generated from blood cells of a different individual and one hESC lines, H9. The representative Western blot and the corresponding quantification is included in the revised manuscript as **Fig. 1i and 1j**

4. Figure 2b lacks loading controls

Response: We agree with the reviewer for not providing the loading controls in Figure 2b (original submission). The MICU1 and Foxd1 levels were normalized with actin protein loading control. The new data is incorporated in the revised manuscript as **supplementary Fig. 1i and 1j**.

5. I can't understand why the authors used a different cellular model (HPMVEC) for ChIP. This must be better discussed.

Response: To ensure the consistency of the phenomenon of Foxd1-mediated MICU1 regulation under hypoxia and the reproducibility, we performed the ChiP and luciferase assay in Human Pulmonary microvascular endothelial cells (HPMVECs). Considering the reviewer's suggestion, the ChIP assay was performed in two iPSC lines: SeV3 and SeV5 exposing them to either normoxia/hypoxia. Our new results are provided in the revised manuscript as **Fig. 2k**. The switch of different cellular models are now well justified in the manuscript text.

6. Panel 2f: in this experimental setup, it looks like there is still a substantial MICU1 expression during hypoxia. Why?

Response: In this experiment, HPMVECs were transfected with the *micu1* promoter constructs for 48h, and the cells were maintained at 20% oxygen and thus overexpressing *micu1*-promoter and luciferase. The decrease in luminescence under hypoxia is a consequence of Foxd1 repression of micu1. Complete absence of luminescence was not observed because of the endogenous Foxd1 repression on the overexpressed *micu1* promoter.

7. Panel 2g: why so many MICU1 bands are present when Foxd1 is silenced?

Response: We wish to notify the reviewer that when there is an overexpression of MICU1, sometimes we tend to see multiple bands that represent pre and mature form of MICU1 as labelled in the revised figure (**Supplemental Figure 1n**).

8. Fig 3a and b show that mitochondrial membrane potential is decreased in SeV3 cells. According to the authors, this is a consequence of MICU1 levels. However, MICU1 downregulation has no effect of organelle membrane potential in other cellular models. Of course, a number of other reasons could account for this difference, and I don't think MICU1 levels are sufficient to explain changes in membrane potential.

Response: We very well agree with the reviewer that the membrane potential ($\Delta\Psi_m$) decrease in hiPSCs could be due to several factors. In the present study, we observe reduced $\Delta\Psi_m$ in hiPSCs and the decrease in the $\Delta\Psi_m$, the driving force, did not affect the MCU-mediated Ca^{2+} uptake. Also, the reconstitution of MICU1 by ectopic expression or knocking down Foxd1, restored $\Delta\Psi_m$ in hiPSCs (**Fig. 3a and 3g**). This explains a possible correlation between MICU1 levels and $\Delta\Psi_m$, at least in part with the perspective of the present study. This new data is given in the revised **Fig. 3a and 3g**. We would also like to bring to light that hiPSCs are glycolytic in nature making them less OXPHOS dependent[1]. The $\Delta\Psi_m$ in these cells is sufficient to drive basal mitochondrial respiration, that is distinct from a

completely OXPHOS dependent cellular models which could also explain the reduced $\Delta\Psi_m$ and future studies are warranted to explain how the glycolytic nature of iPSCs maintain the mitochondrial membrane potential and this is beyond the scope of the current study.

9. Given the impact of MICU1 expression on gene expression (fig 4), I think it would be worth to test the differentiation potential of these cells into different lineages

Response: We thank the reviewer for the thoughtful comment. Since we observed ectopic expression of MICU1 led to an enrichment of EMT pathway proteins in hiPSCs, we asked first if the pluripotent nature of these cells is changed by infecting hiPSCs with AdMICU1. MICU1 expression did not alter the mRNA levels of OCT4, and NANOG (**Supplementary Fig. 3j**) when cells were cultured under pluripotency-maintaining conditions, which was similar to the phenotype of ESCs that lack Snail[2], a transcriptional repressor that controls lineage commitment through EMT regulation.

We next asked whether ectopic expression of MICU1 regulate iPSC-derived early lineage specification. SV20 hiPSCs cultured in pluripotency-maintaining media were infected with AdMICU1. 48 h post-infection, cells were replated and induced for early lineage commitment by separately applying cytokines that specify each of the three germ layers: endoderm, mesoderm, and ectoderm. While the ectopic expression of MICU1 significantly upregulated genes of the endoderm lineage (**Supplementary Fig. 4a**), but had little or no effect on mesoderm or ectoderm genes (**Supplementary Fig. 4b and 4c**). This was not entirely surprising to us. While ectopic expression of MICU1 increased EMT genes, the inductive cues that drive the initial lineage commitment are very dominant and are overriding the impact of ectopic MICU1.

Because EMT has been identified as one of the first steps of cardiac differentiation[3-5] with Ca^{2+} as an essential signal integrator for differentiation of ESCs to functional cardiomyocytes, we followed up with experiments to investigate if ectopic MICU1 expression has an effect on hiPSC-derived cardiomyocyte (iPS-CM) differentiation. Our data show that MICU1 enhanced maturation of newly differentiated iPSC-CMs by both molecular and functional assessment (**Fig. 5**). A similar effect on primary neonatal rat ventricular myocytes was also observed by ectopic MICU1 expression (**Supplementary Fig. 5**). New figures and text are appropriately added in the manuscript to reflect the new data.

Minor points:
1. Page 1: in the introduction paragraph, the reference to the identification of MCU by the Rizzuto group is lacking (refs 8-10)

Response: We apologize for the typographical error. The reference is incorporated appropriately in the revised manuscript.

2. In some panels, expression levels are shown as smoothed traces (1j, 2a, 2c, 2j and 5b), and I think this is misleading. Bar graphs should be used instead, as in the other panels.

Response: Although we originally generated bar graphs, our hypoxia/reoxygenation data suggested that MICU1 could possibly oscillate during normal and stress conditions like fed/fasting state or circadian rhythm to name a few….

Reviewer #2

In this study, Shanmughapriya and colleagues report the novel finding that MICU1 expression is regulated by hypoxia in a FOXD1-dependent manner. They also provide evidence that MICU1 is constitutively down-regulated in IPSCs. Furthermore, as previously reported they show that MICU1 functions as a gatekeeper of mitochondrial Ca++ since in absence of MICU1 mitochondrial Ca++ accumulates and this is associated to increased ROS. Lastly, they claim that cell differentiation is somehow associated to decreased levels of MICU1.
The paper is potentially interesting. However, at this stage it is a descriptive and confusing mosaic of data with no clear take home message. The authors' conclusions are not fully supported by the data as shown. The most solid findings are the ones that confirm previous reports.

Specific Comments:
1. The authors use at least three different cell types (NRVM, HPMVECs and IPSCs), and throughout the paper they keep switching between cell types without providing any rationale but simply increasing the level of confusion and misleading statements.

Response: We apologize for the confusion. To ensure the consistency of this phenomenon and the reproducibility, the assays were performed in different cell types. We believe that this underscores the rigor and reproducibility of our data and does not mislead the reader's view. In line with the reviewer's suggestion, the revised manuscript has now been modified with newly performed assays including the effect of hypoxia and normoxia on MICU1 mRNA and protein abundance (**Fig. 2a-2d**), ChiP assay (**Fig. 2k**), and lineage-specific differentiation (Supplementary **Fig. 4**) in hiPSCs. We used the hiPSCs-derived myocytes (Sev3-CMs) to study the role of MICU1 in cardiomyocyte differentiation and maturation and our new data are provided in the revised manuscript as **Fig. 5**.

2. It is not at all clear why increased Ca++ levels in the mitochondria augments ROS and reduce OCR, at least in hypoxia.

Response: Mitochondrial Ca^{2+} overload is a primary phenomenon that occurs under hypoxic conditions and loss of MICU1 explains a molecular mechanism for the $_mCa^{2+}$ overload (**Supplemental Fig. 2f, and 2i**). Increased Ca^{2+} entry into the mitochondria activates the Ca^{2+}-dependent dehydrogenases that further trigger ETC activity. During the process of increased ETC activity excess electron leakage from complex I and III would react with the available molecular O_2 generating mROS, thus reducing the availability of the end electron acceptor and oxygen consumption. **Supplementary Fig. 3n** represents an increase in the proton leak with a concomitant increase in electron leak that reacts with existing molecular oxygen thus generating mROS.

3. It is completely elusive why hypoxia would cause uncoupling of the oxidative phosphorylation.

Response: Mitochondrial Ca^{2+} overload is a primary phenomenon that occurs under hypoxic conditions and loss of MICU1 explains a molecular mechanism for the $_mCa^{2+}$ overload (**Supplemental Fig. 2f, and 2i**). Increased Ca^{2+} entry into the mitochondria activates the Ca^{2+}-dependent dehydrogenases that further trigger ETC activity. During the process of increased ETC activity excess electron leakage from complex I and III would react with the available molecular O_2 generating mROS, thus reducing the availability of the end electron acceptor and oxygen consumption. **Supplementary Fig. 3n** represents an increase in the proton leak with a concomitant increase in electron leak that reacts with existing molecular oxygen thus generating mROS.

4. More importantly, there is no solid and convincing evidence that decreased levels of MICU1 impair cell differentiation, which in principle would be the most novel aspect of the paper.

Response: We thank the reviewer for the thoughtful comment. Since we observed ectopic expression of MICU1 led to an enrichment of EMT pathway proteins in hiPSCs, we asked first if the pluripotent nature of these cells is changed by infecting hiPSCs with AdMICU1. MICU1 expression did not alter the mRNA levels of OCT4, and NANOG (**Supplementary Fig. 3j**) when cells were cultured under pluripotency-maintaining conditions, which was similar to the phenotype of ESCs that lack Snail[2], a transcriptional repressor that controls lineage commitment through EMT regulation.

We next asked whether ectopic expression of MICU1 regulate iPSC-derived early lineage specification. SV20 hiPSCs cultured in pluripotency-maintaining media were infected with AdMICU1. 48 h post-infection, cells were replated and induced for early lineage commitment by separately applying cytokines that specify each of the three germ layers: endoderm, mesoderm, and ectoderm. While the ectopic expression of MICU1 significantly upregulated genes of the endoderm lineage (**Supplementary Fig. 4a**), but had little or no effect on mesoderm or ectoderm genes (**Supplementary Fig. 4b and 4c**). This was not entirely surprising to us. While ectopic expression of MICU1 increased EMT genes, the inductive cues that drive the initial lineage commitment are very dominant and are overriding the impact of ectopic MICU1.

Because EMT has been identified as one of the first steps of cardiac differentiation[3-5] with Ca^{2+} as an essential signal integrator for differentiation of ESCs to functional cardiomyocytes, we followed up with experiments to investigate if ectopic MICU1 expression has an effect on hiPSC-derived cardiomyocyte (iPS-CM) differentiation. Our data show that MICU1 enhanced maturation of newly differentiated iPSC-CMs by both molecular and functional assessment (**Fig. 5**). A similar effect on primary neonatal rat ventricular myocytes was also observed by ectopic MICU1 expression (**Supplementary Fig. 5**). New figures and text are appropriately added in the manuscript to reflect the new data.

5. The title is an overstatement.

Response: Our new data on lineage-specific differentiation (Supplementary **Fig. 4 and Fig. 5**) makes the new title suitable for the manuscript: “FOXD1-dependent MICU1 expression regulates mitochondrial activity and cell differentiation”.

Reviewer #3:
In this manuscript, Shanmughapriya and co-workers investigate expression and function of Mitochondrial Ca2+ uptake protein 1 (MICU1) in several experimental cell systems.
Respective systems include human fibroblasts and hiPSCs (derived from the corresponding fibroblasts), human pulmonary microvascular endothelial cells (HPMVECs), neonatal rat ventricular myocytes (NRVMs), rodent ventricular myocytes from several embryonic/post-natal stages; gene expression in rodent tissues throughout development was investigated as well.
Specific parts of the manuscript in particular investigations on the interplay of FOXD1 and MICU1 expression and its consequences of Ca2+ ion oscillation in human fibroblast versus hiPSC are interesting. Unfortunately, key data in this part of the paper remain elusive. Other parts are poorly integrated into the context and authors make irrelevant claims based on very poor data sets.

Abstract / Introduction: overall structure and content is difficult to read and understand. E.g. odd list and order in the first sentence: "The morphological changes occurring between early and late embryonic stages coincide with several factors (mechanical load, contractile force generation, blood flow, mechanical stress, and hemodynamics)… " What are the specific differences between: mechanical load versus mechanical stress ?, blood flow versus hemodynamics?

Response: We apologize for the repetitive verbatim in the text that was difficult to read. The manuscript has been revised extensively for a more comprehensive content and structure that is easy to read and comprehend.

Moreover: "The morphological changes occurring between early and late embryonic stages …. facilitate cellular differentiation. Despite these factors, the role of mitochondria in cell morphogenesis during development remains unexplored." A meaningful connection between these sentences remains elusive.

Response: We apologize for the elusive sentence. For better understanding of the reviewer, the connection between the sentences is explained below. The cellular differentiation that occurs between early and late embryonic stages has primarily been attributed to various epigenetic changes. Till date, the role of mitochondria in development has not been explored and our study is the first to highlight the role of mitochondrial calcium during development. The phrase was modified in the revised manuscript.

Figures 1 / 2: GM942 fibroblast and iPS-SeV3 and iPS-SeV5 (resulting from reprograming of these fibroblast) are analyzed if Figures 1k-i and in Figures 2b-c and onwards. Surprisingly, MICU1 mRNA abundance in response of to normoxia/hypoxia treatment, which is presented in the "intercalated". Figure 2a, is not tested in fibroblasts/ iPS but analyzed in primary cardiomyocytes (NRVMs). Notably, the unexpected (and inappropriate) switch in cell systems is not even mentioned in the main text but only hidden in figure legends. This is strange and the expression of MICU1 (on the RNA and protein level) in response to normoxia/hypoxia must be investigated in GM942 and iPS-SeV3/ iPS-SeV5, respectively to make the study conclusive.

Response: Taking reviewer's concern in to consideration we have now quantified the MICU1 mRNA levels and protein abundance in hiPSCs using qRT PCR and Western blot respectively and new data incorporated as **Fig. 2a-2d**. In addition, the levels of Foxd1 mRNA and protein abundance were quantified and our new results strengthen our hypothesis of an inverse regulation of MICU1 and Foxd1 during normoxia/hypoxia (**Fig. 2e-2g**) exhibiting strong evidence for Foxd1-mediated transcriptional regulation of MICU1 during hypoxia.

Furthermore, authors are presenting 1) NRVMs data in Fig. 2a, 2) GM942 and iPS-SeV3/ iPS-SeV5 data in Fig. 2b-c, and 3) HPMVECs for ChIP-analysis and other assays in Fig 2d-j. This heterogeneous intermingling of different cell systems and data is confusing and not well justified in the manuscript.

Response: We apologize for the confusion. To ensure the consistency of this phenomenon and the reproducibility, the assays were performed in different cell types. We believe that this underscores the rigor and reproducibility of our data and does not mislead the reader's view. In line with the reviewer's suggestion, the revised manuscript has now been modified with newly performed assays including the effect of hypoxia and normoxia on MICU1 mRNA and protein abundance (**Fig. 2a-2d**), ChiP assay (**Fig. 2k**), and lineage-specific differentiation (Supplementary **Fig. 4**) in hiPSCs. We used the hiPSCs-derived myocytes (Sev3-CMs) to study the role of MICU1 in cardiomyocyte differentiation and maturation and our new data are provided in the revised manuscript as **Fig. 5**.

Figure 4 a, b: The induction Ca2+ oscillation in hiPSC by MICU1 overexpression is the most interesting part of the paper. However, if there is such direct relation between FOXD1> (inhibiting MICU1) MICU1 > (inducing Ca2+ oscillation) and Ca2+ oscillation as authors' suggest, it's expected that FOXD1 knockdown by siRNA (Figure 2 g, h) should also re-establish Ca2+ oscillation in hiPSC equivalent to MICU1 gain of function in hiPSC (Figure 4 a, b); this must be demonstrated.

Response: Although we have shown that the knockdown of Foxd1 restored MICU1 expression in hiPSCs and the gatekeeping function of MCU-mediated Ca^{2+} uptake (**Fig. 3g-3k**), as reviewer questioned, we utilized the Foxd1 knockdown hiPSCs to show whether Ca^{2+} oscillatory phenotype in Foxd1 KD hiPSCs phenocopies the ectopic expression of MICU1. The reconstitution of MICU1 by Foxd1 knockdown (**Fig. 4a and 4b**) re-established the $_{i}Ca^{2+}$ oscillatory phenotype corroborating MICU1's function in maintaining cellular Ca^{2+} transients (**Fig. 4c- 4e and Supplementary Fig. 3a and 3b**).

Authors found that: "Also, an epithelial to mesenchymal transition (EMT) pathway proteins, N-Cadherin, Vimentin, MMPs, nuclear b-catenin, and fibronectin were enriched in hiPSCs expressing MICU1 (Supplementary Figure 2b-2d)." If these pathways are substantially modulated by MICU1 expression in hiPSC, it deems surprising that maintenance of the pluripotent phenotype was not disrupted. Therefore, after reconstitution of MICU1 expression in hiPSC, the following assays must be tested and shown: conventional flow cytometry analysis for pluripotency markers (TRA1-60, 1-80, SSEA1,3,4, Nanog, Oct3/4), testing hiPSC proliferation kinetics (compared to untreated controls) immunofluorescence staining of hiPSC colonies. Please also indicate: can these cells being maintained / passaged long term?

Response: We thank the reviewer for the thoughtful comments. We performed new experiment to ask whether the ectopic expression of MICU1 change the pluripotent nature of hiPSCs. Surprisingly, MICU1 expression did not alter the mRNA levels of OCT4, and NANOG (**Supplementary Fig. 3j**) which was similar to the phenotype of ESCs that lack Snail[2], a transcriptional repressor that controls lineage commitment through EMT regulation. Also, EMT transcript Zeb2 was indicated to modulate cell-fate decision during the transformation of ESCs to primary germ layer differentiation[6, 7]. Like gastrulation where most of the cells undergo EMT, we expect ectopic expression of MICU1 in hiPSCs to result in a hybrid EMT that enable cells to identify an external signal and acquire maximum cellular transition. It has been known that a fully transformed mesenchymal fate to be associated with mesoderm development, but neural crest delamination in the ectoderm or partial endoderm formation are also consequences of EMT[4, 5]. Our data on lineage-specific differentiation of the hiPSCs expressing MICU1 (Supplementary **Fig. 4**) strengthen our hypothesis of MICU1 expression enabling a hybrid EMT without losing its pluripotent nature a selection of an early lineage-specific differentiation.

It's also not clear from the paper: was FOXD1 expression in hiPSC monitored in response to MICU1 overexpression?

Response: Foxd1 is a transcription factor that regulates MICU1 expression. Being the upstream signal, we suspected Foxd1 abundance not to be modulated by MICU1 overexpression. But to our surprise, we have shown in the revised manuscript that ectopic expression of MICU1 in hiPSCs to decrease

Foxd1 expression (**Fig. 4a**) which was similar to the inverse regulation of MICU1 and Foxd1 in MICU1/Foxd1 mRNA and protein abundance in tissues collected from different developmental stages (**Figs. 1k, 2h, 2i, and 2j**).

Authors make the following claim: "Reconstitution of MICU1 either by Foxd1 knock down or ectopic expression restores the periodic cytosolic Ca2+ oscillations that is a prerequisite for cellular differentiation." If this hypothesis holds true, it's expected that Ca2+ oscillations in human pluripotent stem cells (hPSC) is induced early during differentiation. To confirm their data / hypothesis the following is required: 1) monitoring the lack of Ca2+ oscillation at the pluripotent state in additional hiPSC and hESC lines, 2) induction of Ca2+ oscillation should be observable shortly upon induction of undirected (e.g. via EB formation in FCS media) or lineage specific differentiation (e.g. using published protocols for Endo,- Meso-, and Ecto-dermal derivatives) of hiPSC.

Response: We thank the reviewer for this thoughtful comment.

(1) To substantiate our hypothesis, we used two clonal hiPSC lines (SeV3 and SeV5) generated from skin fibroblasts of one individual, one hiPSC line (SV20) generated from blood cells of a different individual and one hESC lines, H9. Western blot and qRT PCR analysis confirmed the absence of MICU1 protein and mRNA abundance in these hiPSC clones (**Figs. 1i, 1j, and 1l**). We performed $_{i}Ca^{2+}$ transients in these hiPSC clones with (ectopic expression or Foxd1 KD) or without MICU1 expression (**Fig. 4c-4e and Supplementary Fig. 3a and 3b**). Periodic $_{i}Ca^{2+}$ transients are absent in all the above used pluripotent cells. The reconstitution of MICU1 re-established the $_{i}Ca^{2+}$ oscillatory phenotype corroborating MICU1's function in maintaining cellular Ca^{2+} transients (**Fig. 4c-4e and Supplementary Fig. 3a and 3b**).

(2) As exactly highlighted by the reviewer we do observe the induction of Ca^{2+} oscillation in lineage specific differentiation of hiPSCs. Additionally, we observed ectopic expression of MICU1 (**Fig. 4f**) cause a leftward shift (early) in the induction of Ca^{2+} oscillation (**Fig. 4g-i**). The ectopic expression of MICU1 resulted in a significant difference in the amplitude of Ca^{2+} oscillation in mesoderm and endoderm corroborating early lineage specific differentiation in hiPSCs expressing MICU1, while the ectodermal Ca^{2+} oscillation remain unaffected (**Fig. 4g-i**).

Figure 5 A: The immunofluorescence pictures comparing primary CMs with / without MICU1 overexpression are subjective and non-informative; relevant conclusion on sarcomere structures cannot be made from these pictures. Figure 5B: Quantifying the fluorescence intensity and showing it via an "oscillation patterns" is nonsense with respect to the applied assay ology.
Authors: "Expression of MICU1 (AdMICU1) in E9.5, and P0 caused a significant increase in the number of Z-bands compared to E9.5 and P0 WT controls (Figure 5a and b)." It remains elusive which assays have been applied to support this claim. Overall, this is the weakest part of the manuscript. It is poorly interlinked with other parts of the paper and should be removed as it is far-fetched and claims are not supported by relevant data.

Response: As suggested by the reviewer figure 5 was very preliminary in the originally submitted manuscript. We have strengthened this part of the manuscript by measuring the mitochondrial bioenergetics and periodic cytosolic Ca^{2+} cycling in NRVMs (**Supplementary Fig. 5**) and SeV3-derived myocytes (**Fig. 5**). Additionally, qPCR analysis confirmed directed cardiac differentiation of hiPSCs expressing MICU1, where expression of genes encoding cardiac transcription factor, Mef2c (**Fig. 5f**) and cardiac structural and contractile proteins: MYH6 and MYH7 (**Fig. 5g**) were significantly modulated compared to hiPSCs-CMs. The cardiac transcript profile of the hiPSCs-CMs expressing MICU1 was like the maturing myocytes [8,9,10]. Also, the ectopic expression of MICU1 markedly increased the contribution of fatty acid oxidation to overall cardiac energetics of hiPSCs-CMs (**Fig. 5j**) approaching levels observed in myocytes isolated from P5 (**Supplementary Fig. 5g-5k**). The increase in fatty acid oxidation was paralleled by the decrease in glucose oxidation (**Fig. 5h and 5i**). Our data provides an experimental confirmation to molecularly define the mechanism of fetal myocyte maturation coupled with mitochondria's ability to maintain $_{c}Ca^{2+}$ transients and cardiac regeneration.

At the end of the paper it is stated that: “We establish that the differential expression of MICU1 is essential in maintaining developmental plasticity, thereby facilitating adaptation to stress conditions and to allow a well-integrated tissue repair response.” In view of this reviewer, this is a massive overstatement based on the data basis.

Till date, the role of mitochondria in development has not been explored and our study is the first to highlight the role of mitochondrial calcium during development. So, the present study will be a lead for many upcoming work linking mitochondrial calcium signaling and cellular differentiation and development. With our newly added data on lineage-specific differentiation and myocyte maturation, the conclusions reflect the data presented in the current manuscript. However, per the reviewer’s suggestion, we have reworded the conclusion in the discussion section of the text.

REFERENCES

1. Teslaa, T. & Teitell, M.A. Pluripotent stem cell energy metabolism: an update. *EMBO J* **34**, 138-153 (2015).
2. Lin, Y. *et al.* Snail1-dependent control of embryonic stem cell pluripotency and lineage commitment. *Nat Commun* **5**, 3070 (2014).
3. Kovacic, J.C., Mercader, N., Torres, M., Boehm, M. & Fuster, V. Epithelial-to-mesenchymal and endothelial-to-mesenchymal transition: from cardiovascular development to disease. *Circulation* **125**, 1795-1808 (2012).
4. Thiery, J.P., Acloque, H., Huang, R.Y. & Nieto, M.A. Epithelial-mesenchymal transitions in development and disease. *Cell* **139**, 871-890 (2009).
5. Thiery, J.P. & Sleeman, J.P. Complex networks orchestrate epithelial-mesenchymal transitions. *Nat Rev Mol Cell Biol* **7**, 131-142 (2006).
6. Chng, Z., Teo, A., Pedersen, R.A. & Vallier, L. SIP1 mediates cell-fate decisions between neuroectoderm and mesendoderm in human pluripotent stem cells. *Cell Stem Cell* **6**, 59-70 (2010).
7. Goossens, S. *et al.* The EMT regulator Zeb2/Sip1 is essential for murine embryonic hematopoietic stem/progenitor cell differentiation and mobilization. *Blood* **117**, 5620-5630 (2011).
8. Ronaldson-Bouchard, K. *et al.* Advanced maturation of human cardiac tissue grown from pluripotent stem cells. *Nature* **556**, 239-243 (2018).
9. Uosaki, H. *et al.* Transcriptional Landscape of Cardiomyocyte Maturation. *Cell Rep* **13**, 1705-1716 (2015).
10. Yang, X., Pabon, L. & Murry, C.E. Engineering adolescence: maturation of human pluripotent stem cell-derived cardiomyocytes. *Circ Res* **114**, 511-523 (2014).

<u>REVIEWERS' COMMENTS:</u>

Reviewer #1 (Remarks to the Author):

The authors successfully addressed my original criticisms. I think the paper is now significantly improved and it deserves publication.

Reviewer #2 (Remarks to the Author):

In this revised manuscript, the authors have satisfactorily addressed reviewers' comments and concerns.

Reviewer #3 (Remarks to the Author):

Thank you for your comprehensive response to reviewers questions. The manuscript has been substantially improved.
